# RheumQuest: A Gamified Approach to Musculoskeletal Education

**DOI:** 10.15766/mep_2374-8265.11587

**Published:** 2026-03-25

**Authors:** Rob A. Yates, Katherine E. Twist, Brian P. Higgins

**Affiliations:** 1 Medical Student, University of Kentucky College of Medicine; 2 Associate Professor, Department of Internal Medicine, University of Kentucky College of Medicine; 3 Associate Professor, Department of Microbiology, Immunology, and Molecular Genetics, University of Kentucky College of Medicine

**Keywords:** Gamification, Games, Musculoskeletal System, Rheumatology, Pathology, Pharmacology & Toxicology

## Abstract

**Introduction:**

In response to challenges in musculoskeletal education, we developed RheumQuest, a flashcard-style board game designed to reinforce application of clinical presentations to diagnosis and initial management for medical students. This approach seeks to address gaps in traditional teaching methods, offering a dynamic and effective tool for medical students in musculoskeletal education.

**Methods:**

RheumQuest was implemented as a required, formative 1-hour session during the internal medicine clerkship. In this hour-long activity, third-year medical students participated in a competitive small-group session, utilizing a game board and flashcard-style playing cards. Teams advanced by answering questions correctly, with the first team reaching the board's end declared the winner. Pre- and postsession surveys, along with a multiple-choice test, assessed the effectiveness of the intervention on student confidence and knowledge acquisition.

**Results:**

A total of 125 students participated in this educational innovation, with all 125 participants completing both surveys (response rate 100%). Medical knowledge scores (maximum 10 points per block) increased by an average of 0.36 points, from a mean score of 7.47 presession to 7.82 post session (*p* = .009). Confidence in pharmacology, diagnosis, and management all improved post session (each *p* < .001). Students expressed high satisfaction through a qualitative analysis, as indicated by positive ratings and an opportunity for written feedback.

**Discussion:**

This educational game successfully enhanced student knowledge and confidence in musculoskeletal diagnosis, pharmacology, and management during an engaging session. This intervention is cost-effective, scalable, easily replicable, and readily implementable in other institutions.

## Educational Objectives

By the end of this activity, learners will be able to:

1. Identify the most likely musculoskeletal diagnosis from a focused list (inflammatory arthritis, gout/pseudogout, osteoarthritis, septic arthritis, common soft-tissue conditions) based on a brief clinical presentation.
2. Identify appropriate first-line treatments for the above musculoskeletal diseases and state the primary therapeutic mechanism or key adverse-effect consideration.
3. Demonstrate greater self-reported confidence in forming differential diagnoses and proposing initial management plans for common musculoskeletal conditions.

## Introduction

Rheumatology and the musculoskeletal system are often perceived by medical students as particularly challenging areas of medicine. This difficulty likely arises from the involvement of multiple organ systems, the breadth of disease presentations, and the sheer number of differing pathologies and physiologic processes. Learning is further complicated by the numerous drug classes used in treatment, each with distinct mechanisms of action and adverse effects, making both pharmacologic and therapeutic decision-making difficult for learners.^1,2^

The use of educational games has gained increasing popularity in medical education over the past several years for their ability to effectively engage students and enhance learning satisfaction.^3–5^ Gamification, the process of applying game design principles to non-game topics, such as teaching pharmacology or pathophysiology, can be a useful strategy for teaching complex subjects.^6^ Research suggests that this approach may also enhance understanding and retention of knowledge.^7^ The foundation of this approach is built on the concept that games require active decision making, which can improve the student's ability to engage with material and strengthen long-term recall.^8^ The versatility of games has enabled their integration across health care education, adding to the educational value for the students and programs alike.^9–12^

Several *MedEdPORTAL* publications have focused on games in medical education, with most featuring jeopardy-style gameplay.^13–15^ However, few publications have described educational use of board games, and currently, there are no board games on *MedEdPORTAL* that aim to teach medical students rheumatology or the musculoskeletal system.^11,16^

To fill this gap, we developed a flashcard-style board game, RheumQuest, for medical students to review different musculoskeletal conditions and offer a formative assessment. The flashcard format supports active recall; repeated gameplay could support spaced learning, defined as the intentional distribution of learning activities over time to enhance long-term retention of knowledge, if implemented across multiple sessions.^17^

## Methods

We developed this educational innovation in response to course-director observations, in which they noted that some students struggle to apply preclinical musculoskeletal content during case-based sessions and related formative multiple-choice assessments. The development team included a medical student (Rob A. Yates), a rheumatology-trained general internist (Katherine E. Twist), and a basic science faculty member (Brian P. Higgins). Development was informed by prior course evaluations and a review of the literature on educational games. We guided the design using principles of active learning and backward design, aligning all game content with existing course educational objectives.^4–8^ To enhance learning outcomes, this educational innovation introduces a new approach to reinforce material and engage students, aiming to address limitations in traditional teaching methods and provide an effective, dynamic tool for musculoskeletal education.

The University of Kentucky College of Medicine employs a systems-based preclinical curriculum that integrates the pathophysiology of disease with pharmacology. A significant focus within the Musculoskeletal and Integumentary System course is the diagnosis and treatment of musculoskeletal conditions, which spans 4 of the course's 5 weeks during the first year of medical school. After completing this course and the remaining preclinical blocks, students progress to third-year clinical clerkships, including the internal medicine clerkship, an 8-week rotation with a half-day each week dedicated to classroom-based learning. As part of this clerkship, students play RheumQuest to reinforce musculoskeletal knowledge. Participation in the RheumQuest session is mandatory for students on the internal medicine clerkship; the activity is intended to be formative and does not contribute to summative grades.

We created the game board using Microsoft Publisher and developed the flashcards using Microsoft PowerPoint. The flashcard content was derived from the preclinical musculoskeletal course materials covered during the course. Topics were selected based on course educational objectives, and all game content was printed on standard-sized cardstock for durability. Cards were printed double-sided (flip on short edge); each printed card contains a matched clinical presentation on the front and the diagnosis/explanation on the back. During PDF layout, reverse sides may appear adjacent to other cards for printing efficiency, but printed and cut cards are matched pairs.

Using the printed game board (Appendix A) and flashcard-style playing cards (Appendix B), students formed groups of 4–6 around the classroom. They took turns drawing cards and answering questions on the cards. For each correct answer, they advanced to the space on the game board matching the card's color; incorrect answers ended the turn without advancement.

The deck also included specialty cards (Character, Curse, and Action cards) with either more challenging questions or specific gameplay instructions. The winning team was the first team to reach the end of the gameboard. After finishing the game, students were encouraged to review any leftover cards or restart the game to ensure all content was covered.

We provided verbal gameplay instructions before the activity, with a handout of written instructions (Appendix C) also provided. One facilitator explained the game, distributed materials, and answered any questions that arose during the session. The facilitator remained available to clarify rules. Facilitator preparation took about 10 minutes, and the game session lasted roughly 1 hour. We also created a facilitator guide (Appendix D) to promote consistency across sessions. The breakdown of the session consisted of 5 minutes for the facilitator to give directions, 10 minutes for the presession test, 35 minutes for gameplay, and 10 minutes for the post session test. Feasibility of the session's duration was not a challenge, as the median time to reach the end of the board varied by group; no group had issues completing the game in the allotted time. However, time constraints can easily be mitigated by estimating the allotted time of each educational session based on deck size.

Each group had 1 game board (Appendix A) and an assortment of playing cards (Appendix B). Game boards were printed in color, taped together, and featured a path of colored squares to track student progress. Each student chose a character token, such as Sir Rheumalot, Knight Arthritus, Dame Cartilena, or Baron Goutfrey.

The playing cards (Appendix B) included 64 content cards and 10 gameplay cards. Content cards reinforced key topics, focusing on 86% diagnostic questions and 14% drug mechanisms/characteristics (e.g., adverse effects). The Curse cards posed challenging, multistep questions on pharmacology, while gameplay cards added actions like skipping turns or using an outside resource.

Content was developed by Katherine E. Twist (a rheumatology-trained general internist) and Rob A. Yates (a medical student), with subject-matter review provided by Brian P. Higgins. Multiple-choice questions were written by Katherine E. Twist and vetted by 3 internal medicine faculty. Facilitators were primarily the above-stated team. However, several sessions were performed by internal medicine educators, who effectively performed the session using only the provided printed materials and the instructions, normally requiring about 10 minutes to read before the session.

All 74 cards were printed on cardstock and cut to size, with decks shuffled and distributed to each group. The facilitator oriented students to the gameplay and remained available to clarify rules. Cards were not distributed for take-home use to prevent feedforwarding.

Student knowledge and satisfaction was measured before and after the activity (Kirkpatrick Level 1 and Level 2).^18^ To measure knowledge (Kirkpatrick Level 2), the team created 20 multiple-choice questions encompassing various musculoskeletal diagnoses that aligned with course educational objectives and game content. The team divided the 20 questions into 2 sets of 10 (block A and block B), with each participant receiving 1 set before the intervention and the other set after the intervention (Appendix E). To account for possible differences in question difficulty between 10-question blocks, students were divided so that half of them completed block A and then block B questions and the other half completed block B and then block A questions. To minimize testing burden within a 1-hour session, knowledge was assessed with 2 counterbalanced 10-item blocks. Because item-level response data were unavailable for this data set, retrospective psychometric analysis (e.g., Cronbach's alpha, item difficulty, point-biserial correlations) could not be performed.

Students’ self-perceived knowledge (Kirkpatrick Level 2) was measured by asking them to rate their confidence on a 5-point Likert scale (1 = *not at all confident*, 5 = *extremely confident*) in responding to the multiple-choice questions before and after the game. For measurement of student satisfaction (Kirkpatrick Level 1), a 4-point Likert scale (1 = *not helpful*, 4 = *very helpful*) was used to gauge how helpful students found various game aspects for learning (Appendix E). A 4-point Likert scale was used for gauging satisfaction, to avoid a neutral midpoint.

Knowledge improvement was analyzed using a paired 2-tailed *t* test, and a 2-tailed *t* test was used to assess differences from pre- to post session by question order (block A–B vs. block B–A groups). Confidence ratings were compared before and after the intervention using a paired *t* test, while helpfulness ratings were analyzed descriptively. All tests used an alpha level of .05 to determine significance.

The team further stratified knowledge question data, analyzing individual changes in scores from pre- to postsession to examine the intervention's impact on participants based on their initial knowledge. This offered insights into knowledge improvement trajectories influenced by students’ baseline understanding.

The University of Kentucky Institutional Review Board reviewed this project and determined it to be exempt educational research (granted on August 24, 2023; No. 89845).

## Results

A total of 125 third-year medical students participated in this activity during their internal medicine rotation throughout the school year. All 125 participants completed both the presession and the post session surveys (response rate 100%).

Knowledge scores improved after participation in the game, from a mean score of 7.47 (of 10) before the intervention to a mean score of 7.82 (of 10) after the intervention (*p* = .05). No effect was observed based on the order in which questions were administered (*p* = .92). However, individual scores increased significantly from pre- to post session (*p* < .01).

Further analysis revealed that the intervention had the greatest impact on students whose initial knowledge scores were lower. Those with presession knowledge scores ranging from 3 to 6 (of 10) showed an average increase in score of 1.71 points, whereas students with higher initial scores (ranging from 7 to 10 of 10) showed an average decrease in score of 0.09 points. Given the brief 10-item format and narrow scoring range, the absolute mean increase should be interpreted as directional improvement rather than evidence of mastery.

Students’ confidence in diagnosis (rated on a 5-point Likert scale) also improved, increasing from a pregame mean score of 2.80 to a postgame mean score of 3.21. The 2-tailed paired *t* test comparing scores demonstrated a significant increase in confidence among all participants (*p* < .01). Confidence is a subjective, self-perceived construct and may not map directly to objective knowledge gains; modest increases in confidence after a single session are not unexpected.^19,20^

Student ratings of the activity's helpfulness (measured on a 4-point Likert scale) showed a mean score of 3.3 (of 4) after the activity.

At the end of the session, participants shared feedback, highlighting several recurring themes. Many found the session engaging and enjoyable, with comments like “fun” and “a refreshing way to learn,” contrasting it with traditional education methods. The group-based format was appreciated for its collaborative aspect. As 1 student remarked, “Talking through the diagnosis with classmates was helpful.” The low-stress environment fostered a supportive atmosphere that encouraged active participation. Additionally, the session reinforced key skills like active recall and pattern recognition while covering a broad range of content, allowing students to identify knowledge gaps and areas for review. Overall, the feedback reflected a strong appreciation for the session's interactive, comprehensive approach.

## Discussion

This interactive educational activity offers an innovative approach to reinforcing students’ knowledge of musculoskeletal diseases and their management. Based on both quantitative and qualitative analysis, this intervention proved effective in meeting the course's educational objectives while actively engaging participants. Our results demonstrated improvements in students’ performance on knowledge-based questions, an increase in confidence, and high participant satisfaction. Consequently, this approach appears to be a valuable tool for reinforcing knowledge and engaging students in an interactive, nontraditional learning environment.

The intervention was also identified as a low-cost, interactive educational activity that requires minimal time and effort to prepare. It is time-efficient, fitting comfortably into a 1-hour session. Feedback indicated that participants thoroughly enjoyed this unique, low-stakes approach to learning, with positive comments including describing it as “super fun.” Given the positive student engagement and improved knowledge outcomes, the intervention successfully fostered student growth and development while achieving its educational objectives.

Notably, the intervention had the greatest impact on lower-performing students, which is particularly beneficial given its goal of directly enhancing students’ knowledge of musculoskeletal topics in rheumatology. Lower-scoring students benefited the most, with higher-scoring students showing little net improvement, likely due to their already high baseline knowledge, leaving less margin for growth. This highlights the intervention's potential for identifying and benefiting students who may be struggling or on the lower end of the knowledge spectrum.

Several limitations of this educational innovation should be acknowledged. The team did not assess long-term knowledge retention, so it is unclear if the educational gains were sustained over time. Additionally, while the game aligned well with the team's curriculum, its content may not perfectly fit other institutions, potentially limiting generalizability. Also, the assessments contained only 10 items, and item-level data were unavailable; the educational innovation could not present internal-consistency or item-discrimination metrics, leading to the inability to include psychometric evaluation. Finally, this educational innovation's single-institution setting and relatively small sample size may restrict the broader applicability of the findings, though these limitations were considered minor relative to the educational benefits observed.

This game-based approach proved to be a simple, cost-effective way to engage students, requiring minimal preparation, limited to printing and cutting boards and game cards. It was well-received by students (Kirkpatrick Level 1) and effective in enhancing learning (Kirkpatrick Level 2). With minimal adaptation, this activity could be implemented at other institutions, as materials are easy to prepare and scalable for different group sizes.

Looking ahead, it will be useful to explore additional educational gamification opportunities to extend the success of this intervention into other areas of medical education. Future work should also investigate spaced implementations (repeated sessions across the clerkship), which may produce larger, more durable knowledge and confidence gains. This approach has proven effective for engaging learners and reinforcing content, while also being cost effective and requiring minimal preparation. By continuing to develop student-centered interactive games, the team aims to enhance medical education through tools that support diverse learning styles, ultimately fostering a more inclusive and effective learning environment for all students.

## Appendices


RheumQuest Board.pdfRheumQuest Cards.pptxRheumQuest Instructions.docxFacilitator Guide.docxPre- and Posttest with Answer Key.docx
*All appendices are peer reviewed as integral parts of the Original Publication.*
