# Supplementary material for: RheumQuest: A Gamified Approach to Musculoskeletal Education
Source: MedEdPORTAL. 2026 Mar 25;22:11587. doi: 10.15766/mep_2374-8265.11587 (PMC13013083; doi:10.15766/mep_2374-8265.11587)
Supplement: Supplementary file 1 — RheumQuest Board.pdfRheumQuest Cards.pptxRheumQuest Instructions.docxFacilitator Guide.docxPre- and Posttest with Answer Key.docx [file mep_2374-8265.11587-s001.zip › B. RheumQuest Cards.pptx]

## Slide 1
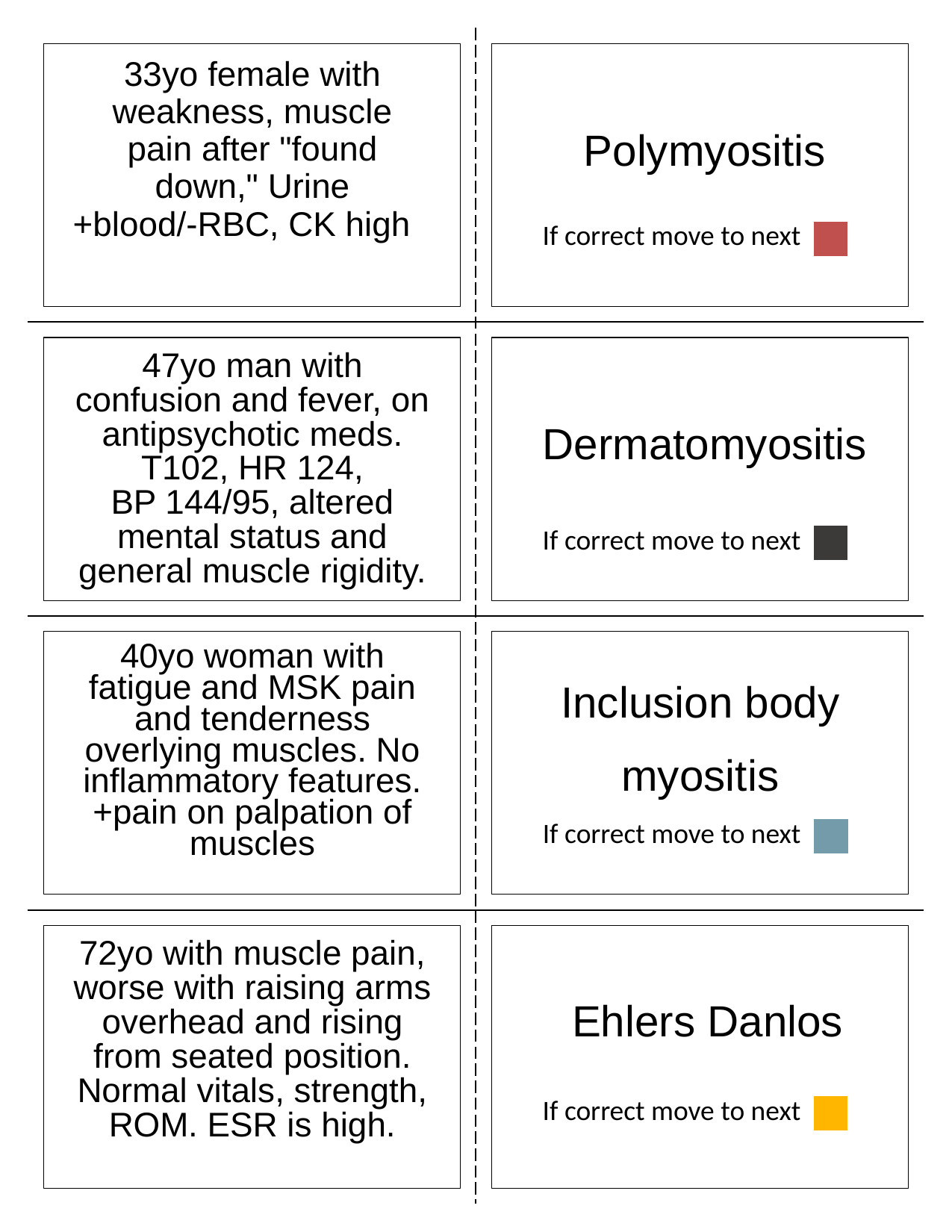

33yo female with weakness, muscle pain after "found down," Urine
+blood/-RBC, CK high
Polymyositis
If correct move to next
47yo man with confusion and fever, on antipsychotic meds. T102, HR 124,
BP 144/95, altered mental status and general muscle rigidity.
Dermatomyositis
If correct move to next
40yo woman with fatigue and MSK pain and tenderness overlying muscles. No inflammatory features. +pain on palpation of muscles
Inclusion body myositis
If correct move to next
72yo with muscle pain, worse with raising arms overhead and rising from seated position.
Normal vitals, strength, ROM. ESR is high.
Ehlers Danlos
If correct move to next

## Slide 2
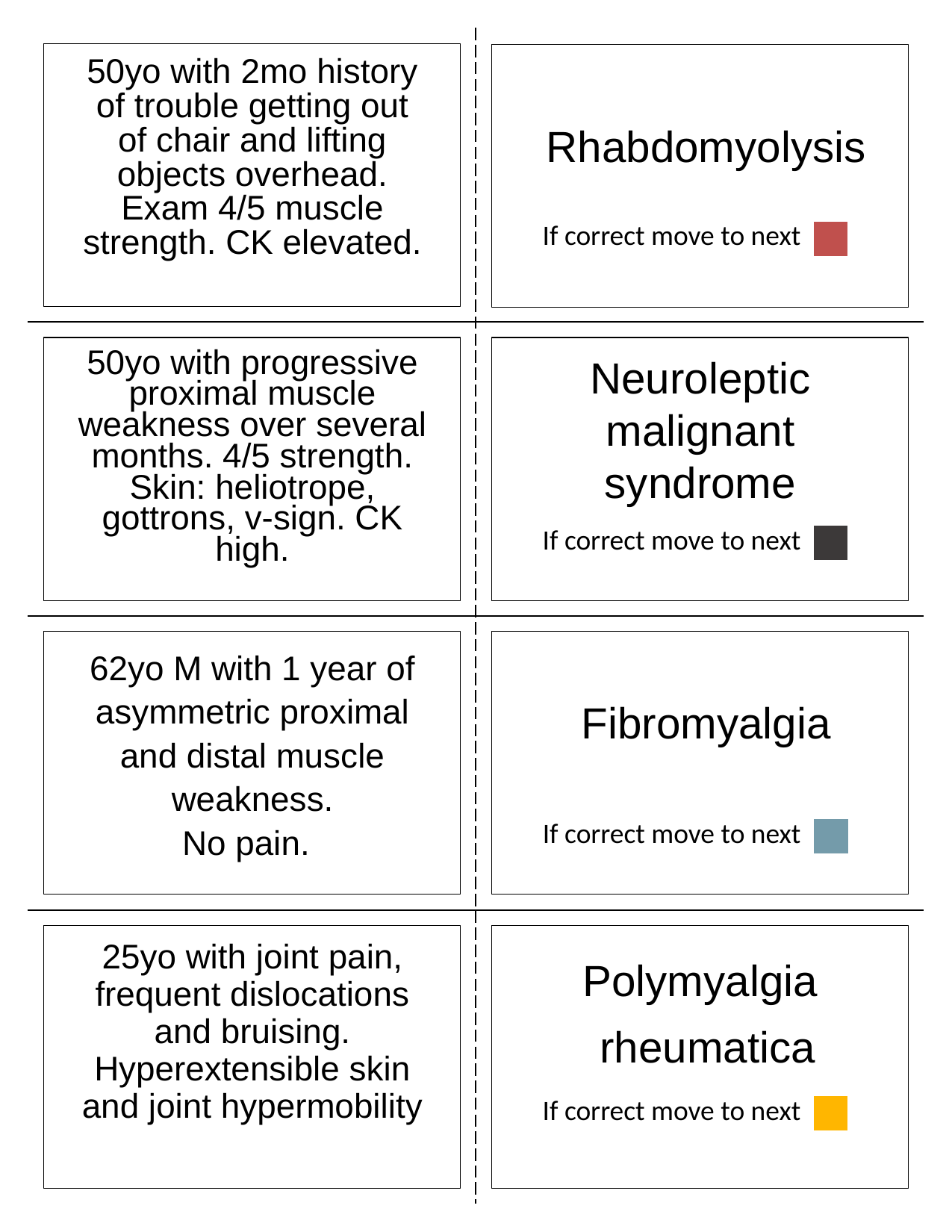

50yo with 2mo history of trouble getting out of chair and lifting objects overhead. Exam 4/5 muscle strength. CK elevated.
Rhabdomyolysis
If correct move to next
50yo with progressive proximal muscle weakness over several months. 4/5 strength. Skin: heliotrope, gottrons, v-sign. CK high.
Neuroleptic malignant syndrome
If correct move to next
62yo M with 1 year of asymmetric proximal and distal muscle weakness.
No pain.
Fibromyalgia
If correct move to next
25yo with joint pain, frequent dislocations and bruising.
Hyperextensible skin and joint hypermobility
Polymyalgia rheumatica
If correct move to next

## Slide 3
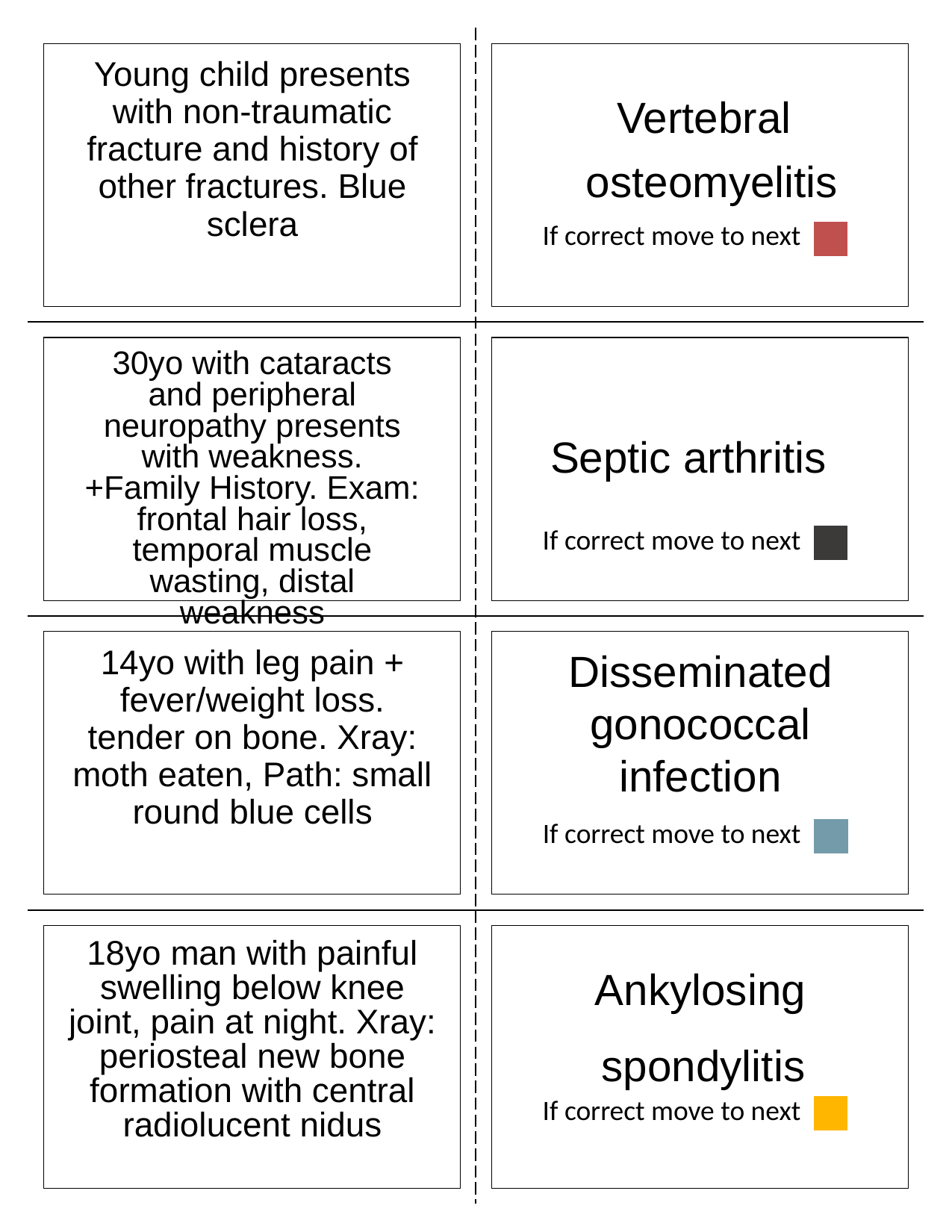

Young child presents with non-traumatic fracture and history of other fractures. Blue sclera
Vertebral osteomyelitis
If correct move to next
30yo with cataracts and peripheral neuropathy presents with weakness.
+Family History. Exam: frontal hair loss, temporal muscle wasting, distal weakness
Septic arthritis
If correct move to next
14yo with leg pain + fever/weight loss. tender on bone. Xray: moth eaten, Path: small round blue cells
Disseminated gonococcal infection
If correct move to next
18yo man with painful swelling below knee joint, pain at night. Xray: periosteal new bone formation with central radiolucent nidus
Ankylosing spondylitis
If correct move to next

## Slide 4
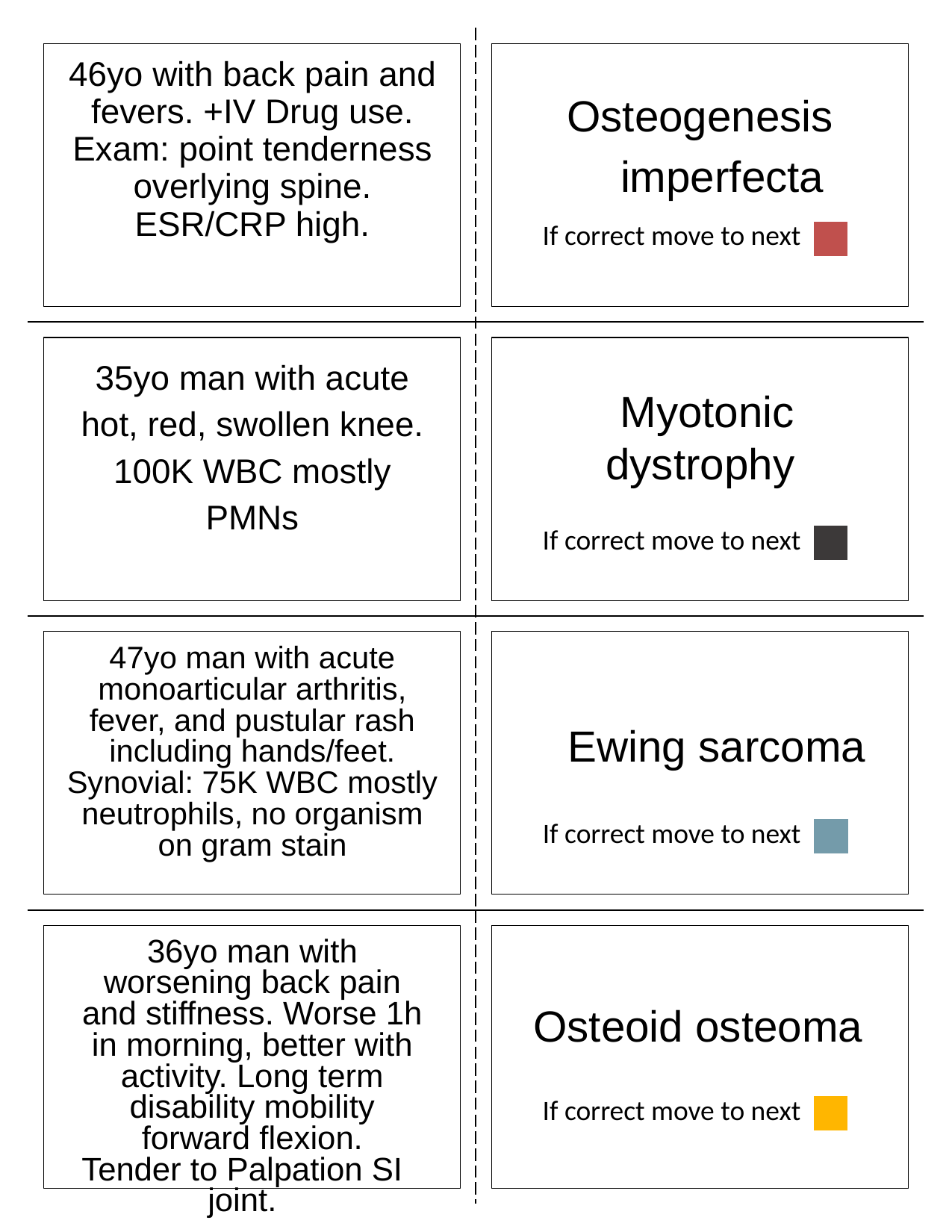

46yo with back pain and fevers. +IV Drug use. Exam: point tenderness overlying spine. ESR/CRP high.
Osteogenesis imperfecta
If correct move to next
35yo man with acute hot, red, swollen knee. 100K WBC mostly PMNs
Myotonic dystrophy
If correct move to next
47yo man with acute monoarticular arthritis, fever, and pustular rash including hands/feet.
Synovial: 75K WBC mostly neutrophils, no organism on gram stain
Ewing sarcoma
If correct move to next
36yo man with worsening back pain and stiffness. Worse 1h in morning, better with activity. Long term disability mobility forward flexion.
Tender to Palpation SI joint.
Osteoid osteoma
If correct move to next

## Slide 5
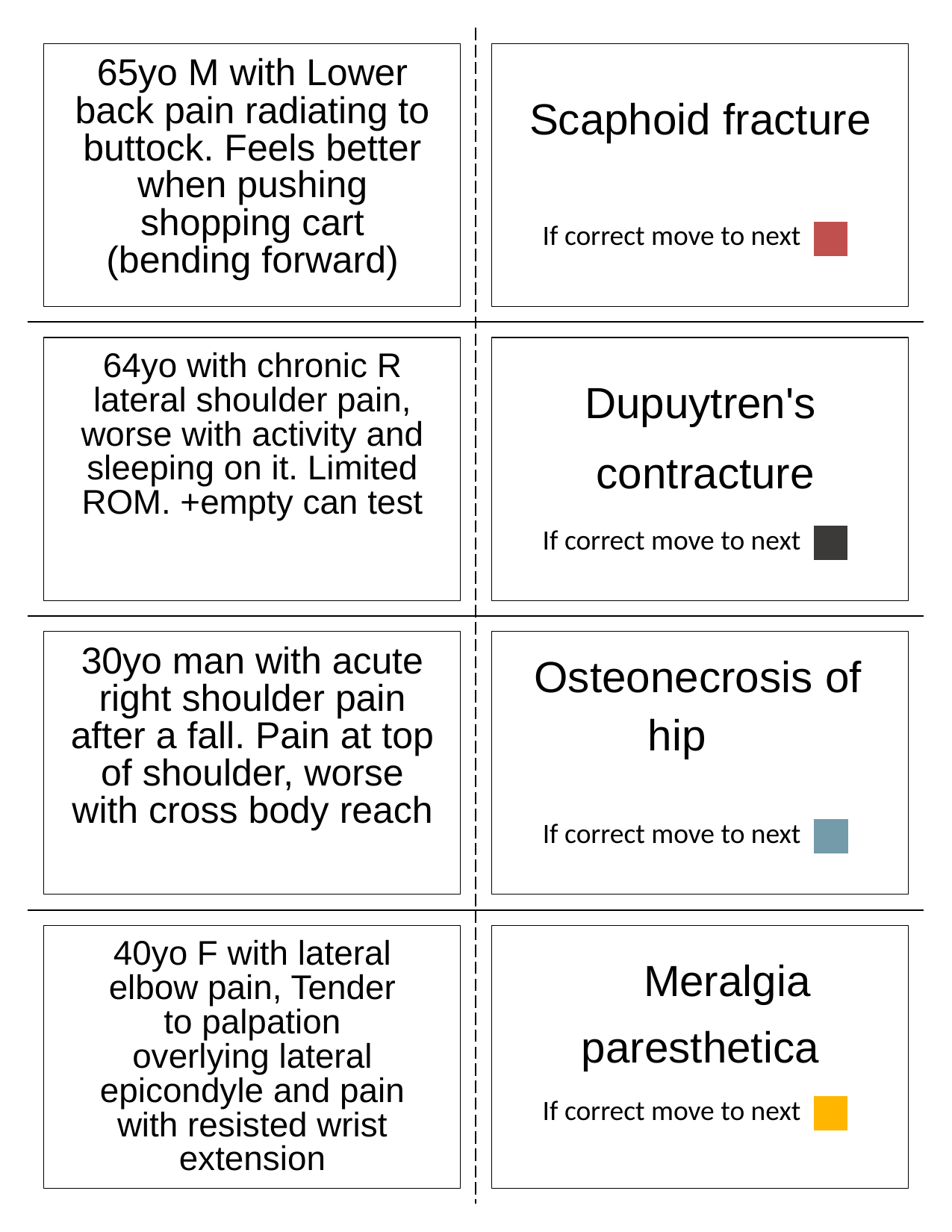

65yo M with Lower back pain radiating to buttock. Feels better when pushing shopping cart (bending forward)
Scaphoid fracture
If correct move to next
64yo with chronic R lateral shoulder pain, worse with activity and sleeping on it. Limited ROM. +empty can test
Dupuytren's contracture
If correct move to next
30yo man with acute right shoulder pain after a fall. Pain at top of shoulder, worse with cross body reach
Osteonecrosis of hip
If correct move to next
40yo F with lateral elbow pain, Tender to palpation overlying lateral epicondyle and pain with resisted wrist extension
Meralgia paresthetica
If correct move to next

## Slide 6
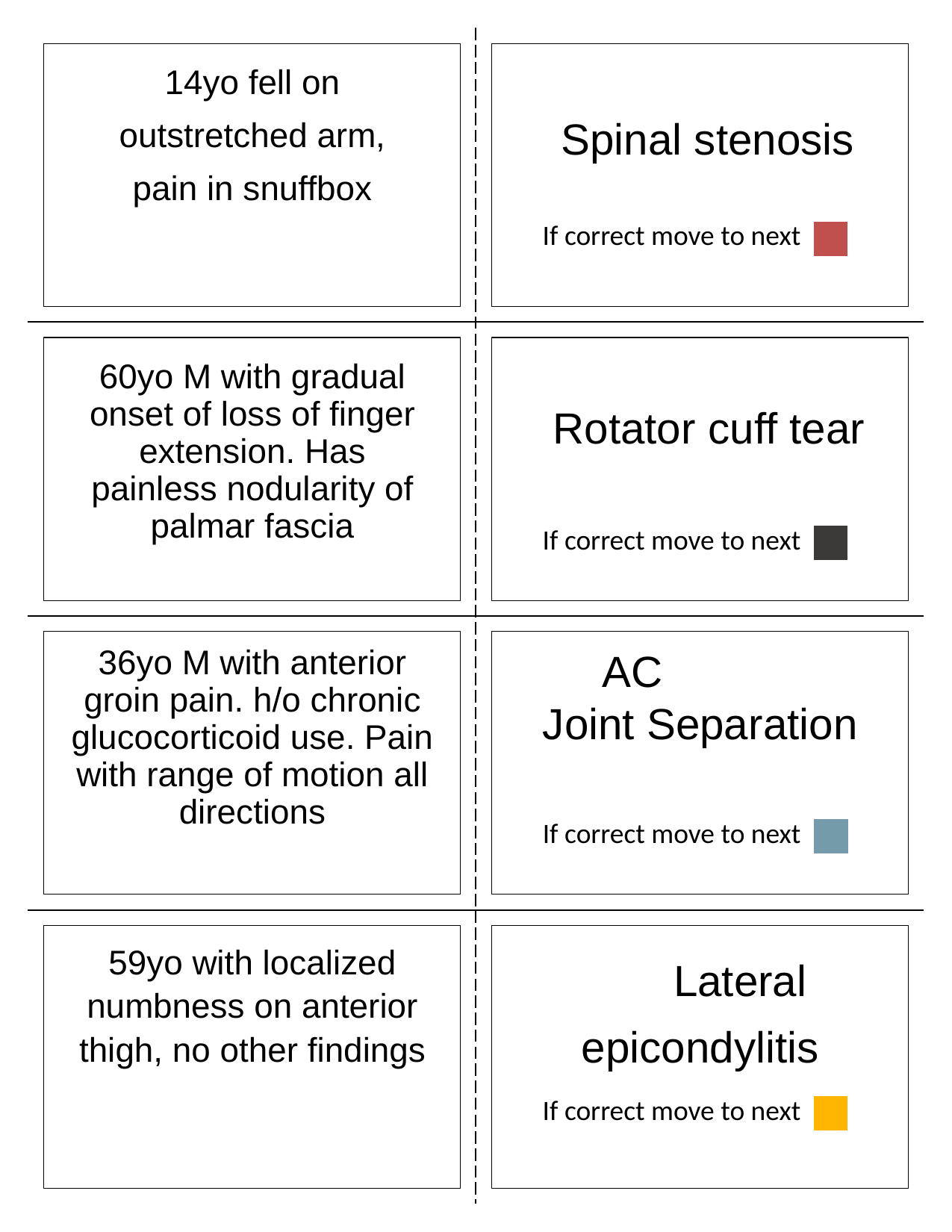

14yo fell on outstretched arm, pain in snuffbox
Spinal stenosis
If correct move to next
Rotator cuff tear
60yo M with gradual onset of loss of finger extension. Has painless nodularity of palmar fascia
If correct move to next
36yo M with anterior groin pain. h/o chronic glucocorticoid use. Pain with range of motion all directions
AC Joint Separation
If correct move to next
59yo with localized numbness on anterior thigh, no other findings
Lateral epicondylitis
If correct move to next

## Slide 7
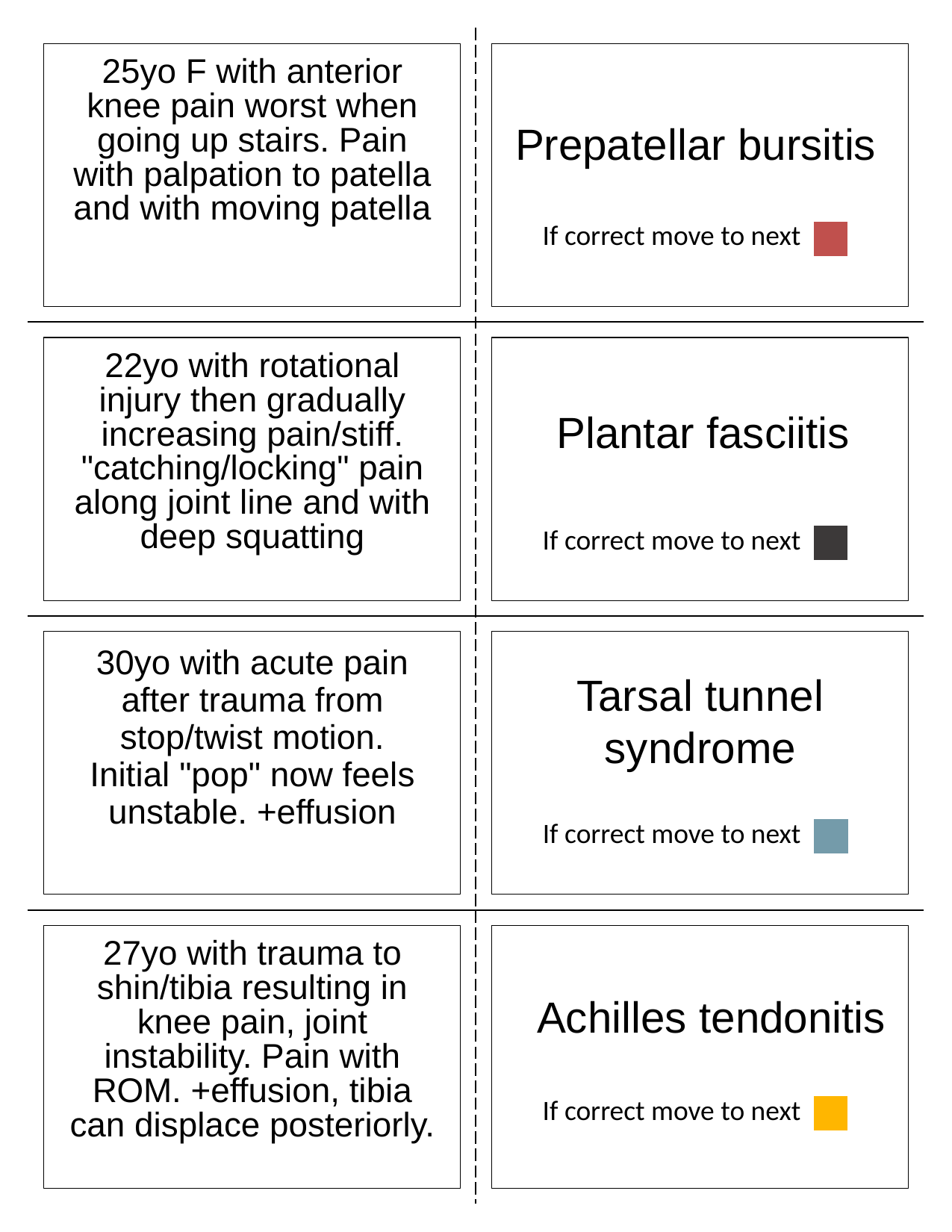

25yo F with anterior knee pain worst when going up stairs. Pain with palpation to patella and with moving patella
Prepatellar bursitis
If correct move to next
22yo with rotational injury then gradually increasing pain/stiff. "catching/locking" pain along joint line and with deep squatting
Plantar fasciitis
If correct move to next
30yo with acute pain after trauma from stop/twist motion.
Initial "pop" now feels unstable. +effusion
Tarsal tunnel syndrome
If correct move to next
27yo with trauma to shin/tibia resulting in knee pain, joint instability. Pain with ROM. +effusion, tibia can displace posteriorly.
Achilles tendonitis
If correct move to next

## Slide 8
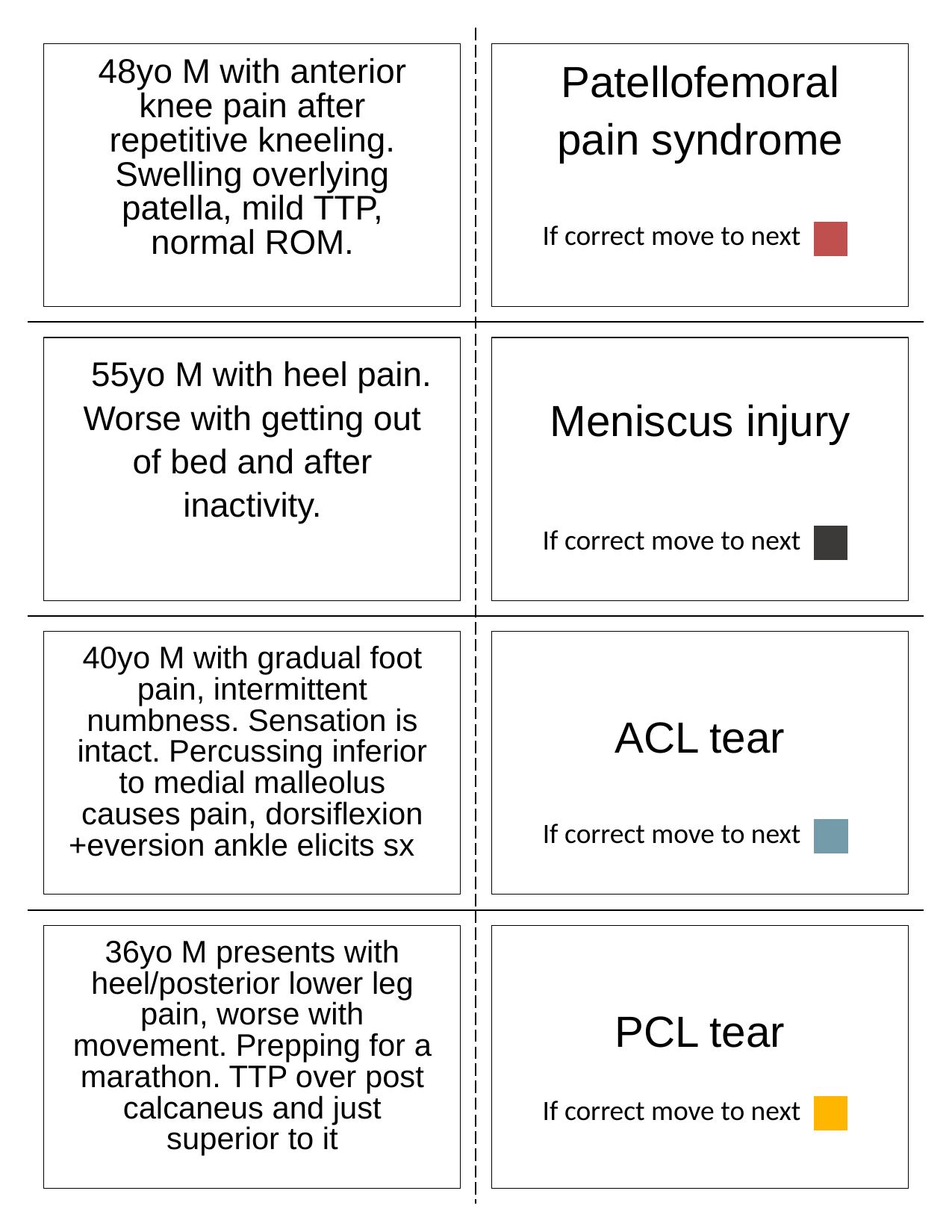

Patellofemoral pain syndrome
48yo M with anterior knee pain after repetitive kneeling.
Swelling overlying patella, mild TTP, normal ROM.
If correct move to next
55yo M with heel pain. Worse with getting out of bed and after inactivity.
Meniscus injury
If correct move to next
40yo M with gradual foot pain, intermittent numbness. Sensation is intact. Percussing inferior to medial malleolus causes pain, dorsiflexion
+eversion ankle elicits sx
ACL tear
If correct move to next
36yo M presents with heel/posterior lower leg pain, worse with movement. Prepping for a marathon. TTP over post calcaneus and just superior to it
PCL tear
If correct move to next

## Slide 9
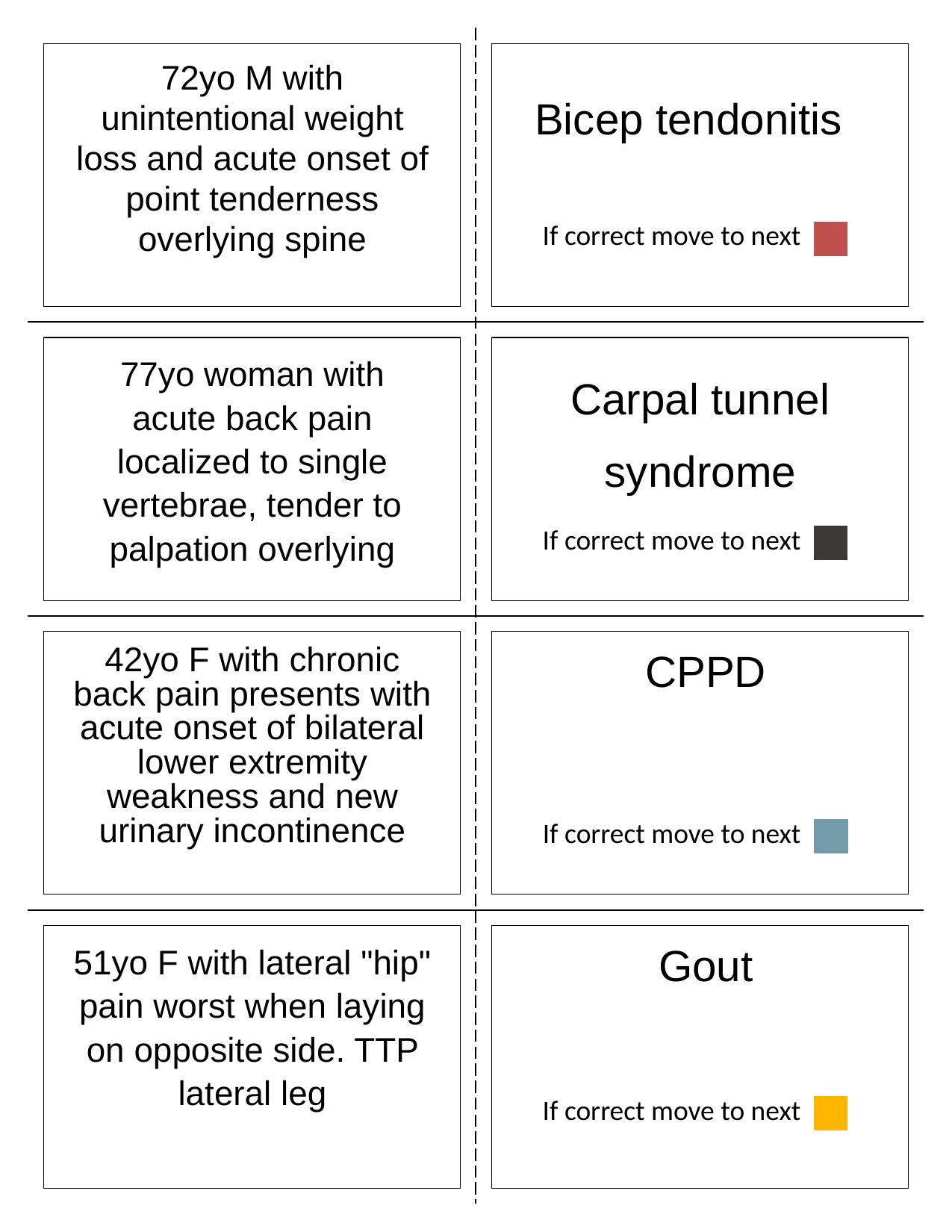

72yo M with unintentional weight loss and acute onset of point tenderness overlying spine
Bicep tendonitis
If correct move to next
77yo woman with acute back pain localized to single vertebrae, tender to palpation overlying
Carpal tunnel syndrome
If correct move to next
42yo F with chronic back pain presents with acute onset of bilateral lower extremity weakness and new urinary incontinence
CPPD
If correct move to next
51yo F with lateral "hip" pain worst when laying on opposite side. TTP lateral leg
Gout
If correct move to next

## Slide 10
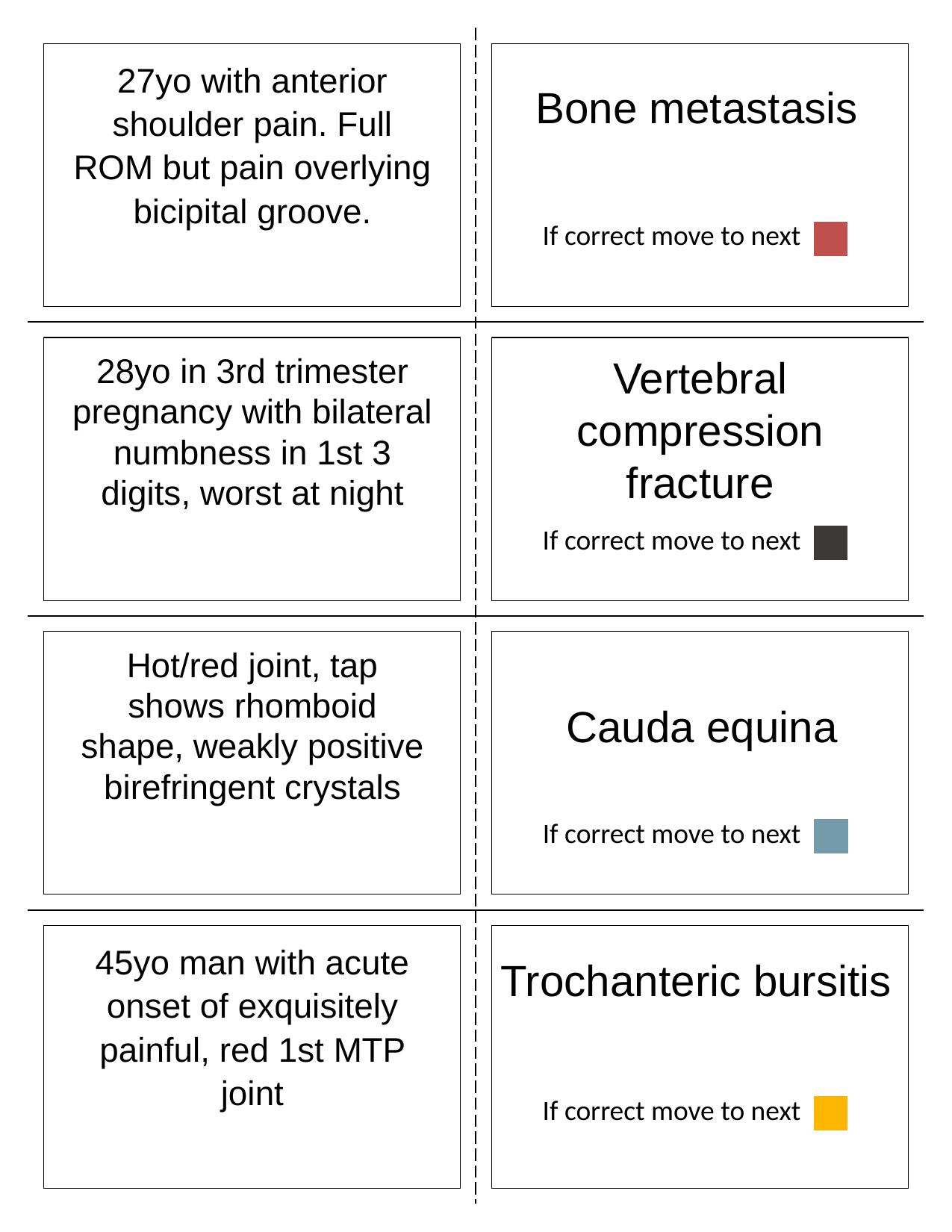

27yo with anterior shoulder pain. Full ROM but pain overlying bicipital groove.
Bone metastasis
If correct move to next
28yo in 3rd trimester pregnancy with bilateral numbness in 1st 3 digits, worst at night
Vertebral compression fracture
If correct move to next
Hot/red joint, tap shows rhomboid shape, weakly positive birefringent crystals
Cauda equina
If correct move to next
45yo man with acute onset of exquisitely painful, red 1st MTP joint
Trochanteric bursitis
If correct move to next

## Slide 11
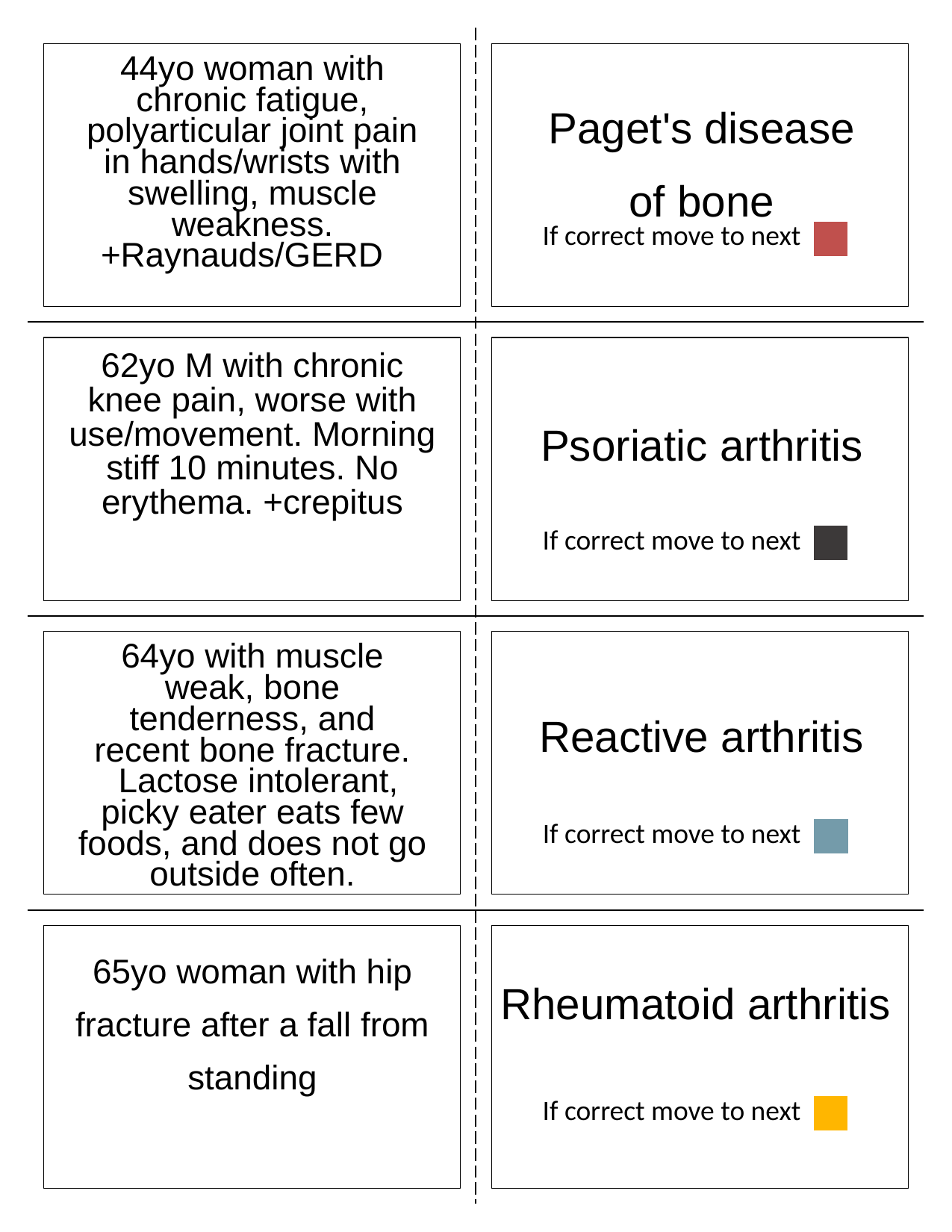

44yo woman with chronic fatigue, polyarticular joint pain in hands/wrists with swelling, muscle weakness.
+Raynauds/GERD
Paget's disease of bone
If correct move to next
62yo M with chronic knee pain, worse with use/movement. Morning stiff 10 minutes. No erythema. +crepitus
Psoriatic arthritis
If correct move to next
64yo with muscle weak, bone tenderness, and recent bone fracture.
Lactose intolerant, picky eater eats few foods, and does not go outside often.
Reactive arthritis
If correct move to next
65yo woman with hip fracture after a fall from standing
Rheumatoid arthritis
If correct move to next

## Slide 12
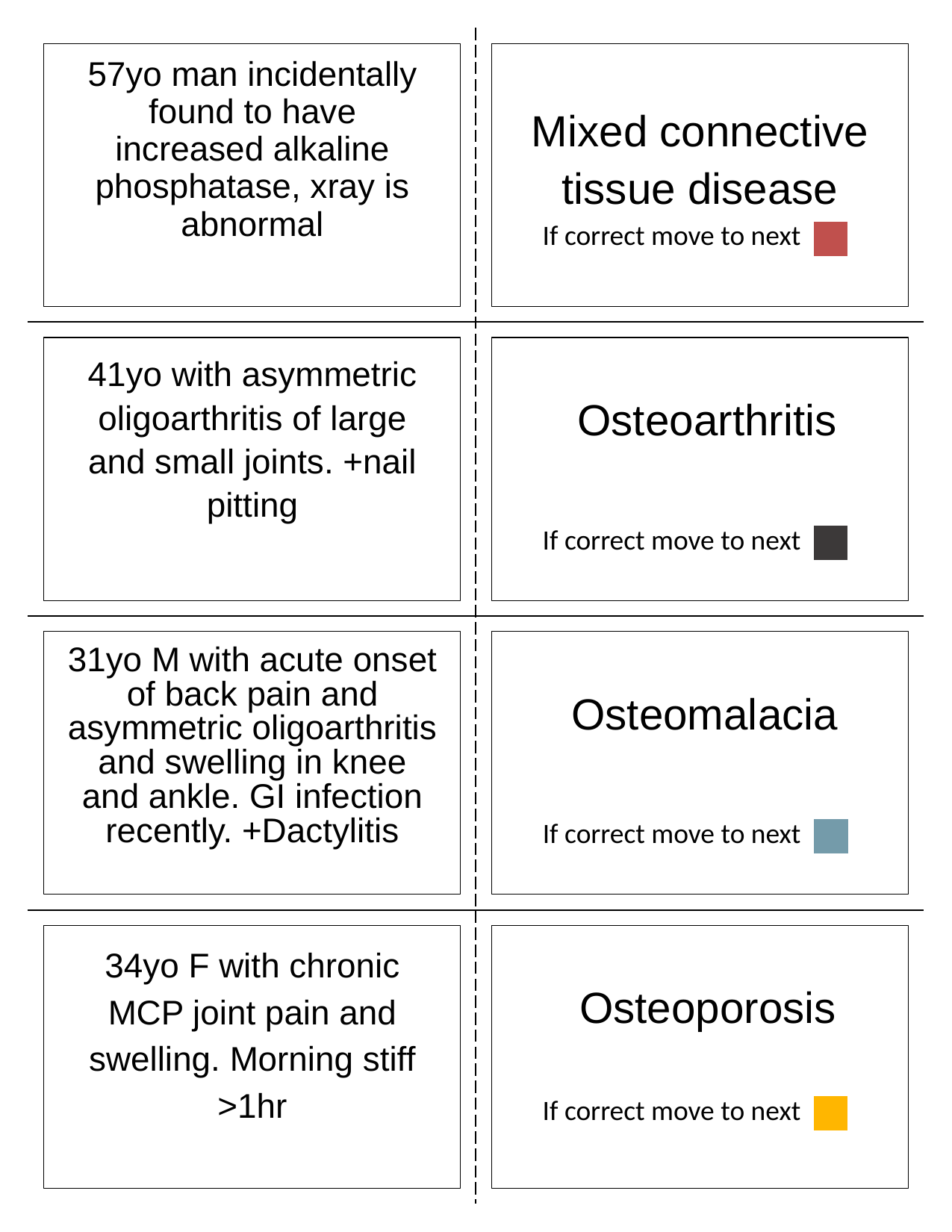

57yo man incidentally found to have increased alkaline phosphatase, xray is abnormal
Mixed connective tissue disease
If correct move to next
41yo with asymmetric oligoarthritis of large and small joints. +nail pitting
Osteoarthritis
If correct move to next
31yo M with acute onset of back pain and asymmetric oligoarthritis and swelling in knee and ankle. GI infection recently. +Dactylitis
Osteomalacia
If correct move to next
34yo F with chronic MCP joint pain and swelling. Morning stiff >1hr
Osteoporosis
If correct move to next

## Slide 13
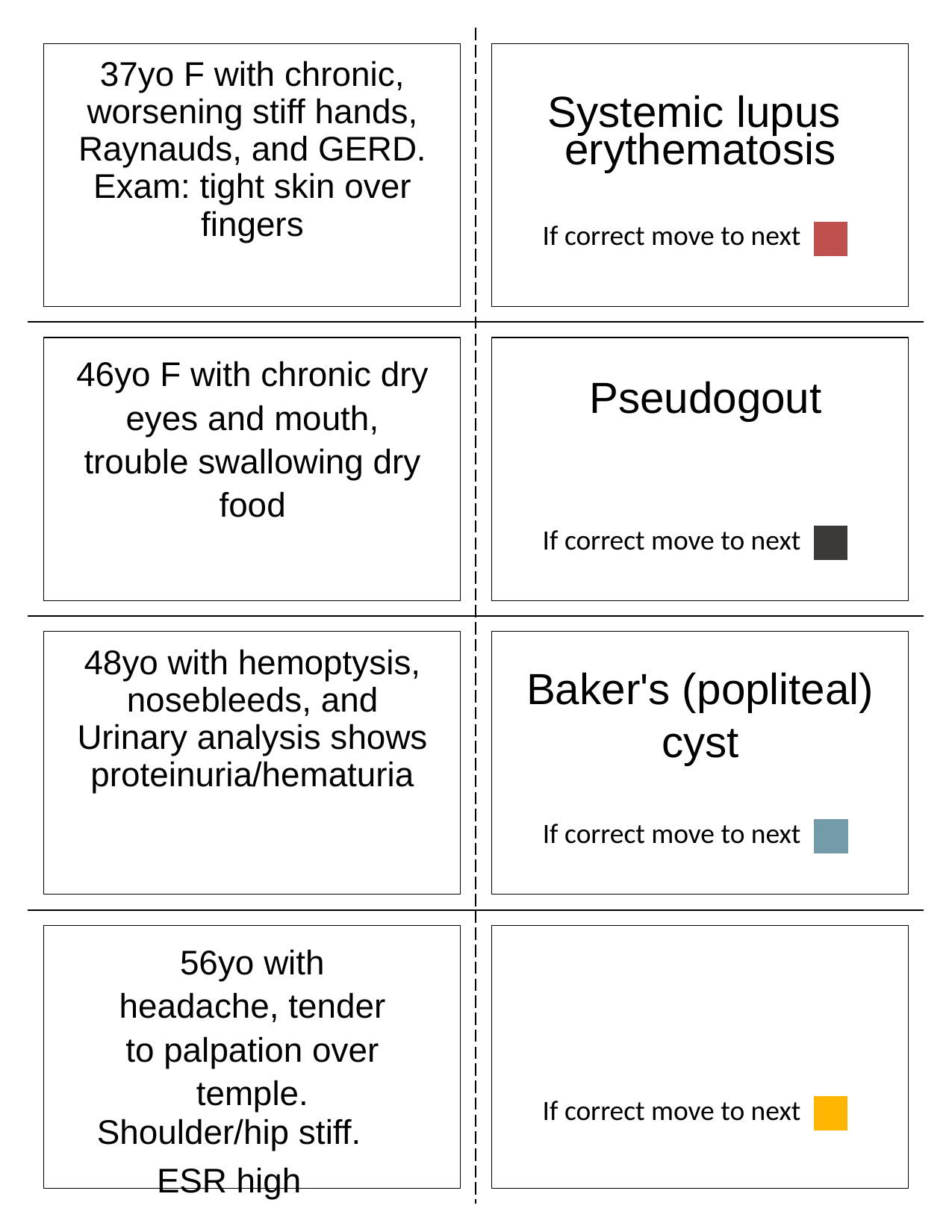

37yo F with chronic, worsening stiff hands, Raynauds, and GERD. Exam: tight skin over fingers
Systemic lupus
erythematosis
If correct move to next
46yo F with chronic dry eyes and mouth, trouble swallowing dry food
Pseudogout
If correct move to next
48yo with hemoptysis, nosebleeds, and Urinary analysis shows proteinuria/hematuria
Baker's (popliteal) cyst
If correct move to next
56yo with headache, tender to palpation over temple.
Shoulder/hip stiff.
ESR high
If correct move to next

## Slide 14
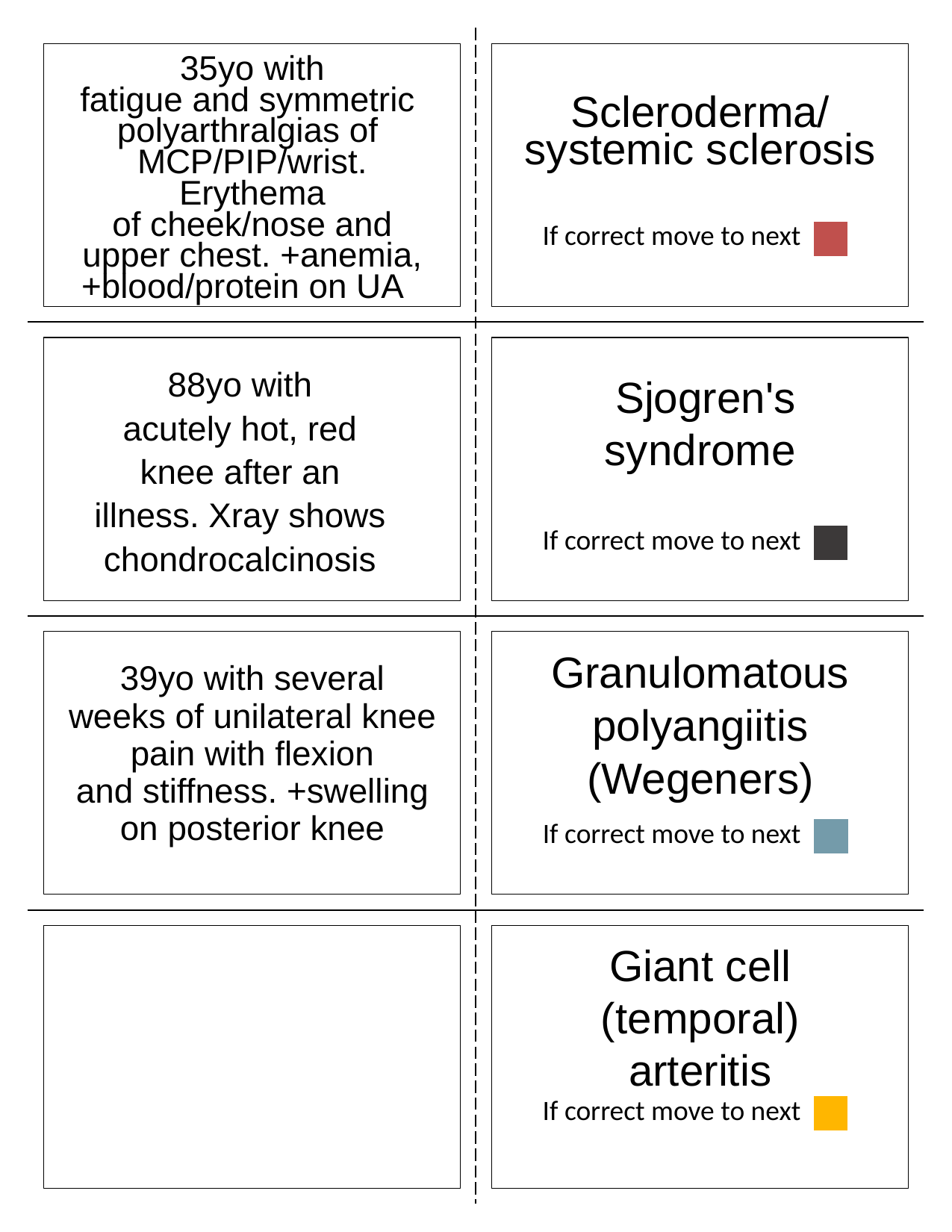

35yo with fatigue and symmetric
polyarthralgias of
MCP/PIP/wrist. Erythema of cheek/nose and upper chest. +anemia,
+blood/protein on UA
Scleroderma/
systemic sclerosis
If correct move to next
88yo with acutely hot, red knee after an illness. Xray shows chondrocalcinosis
Sjogren's syndrome
If correct move to next
Granulomatous polyangiitis (Wegeners)
39yo with several weeks of unilateral knee pain with flexion and stiffness. +swelling on posterior knee
If correct move to next
Giant cell (temporal) arteritis
If correct move to next

## Slide 15
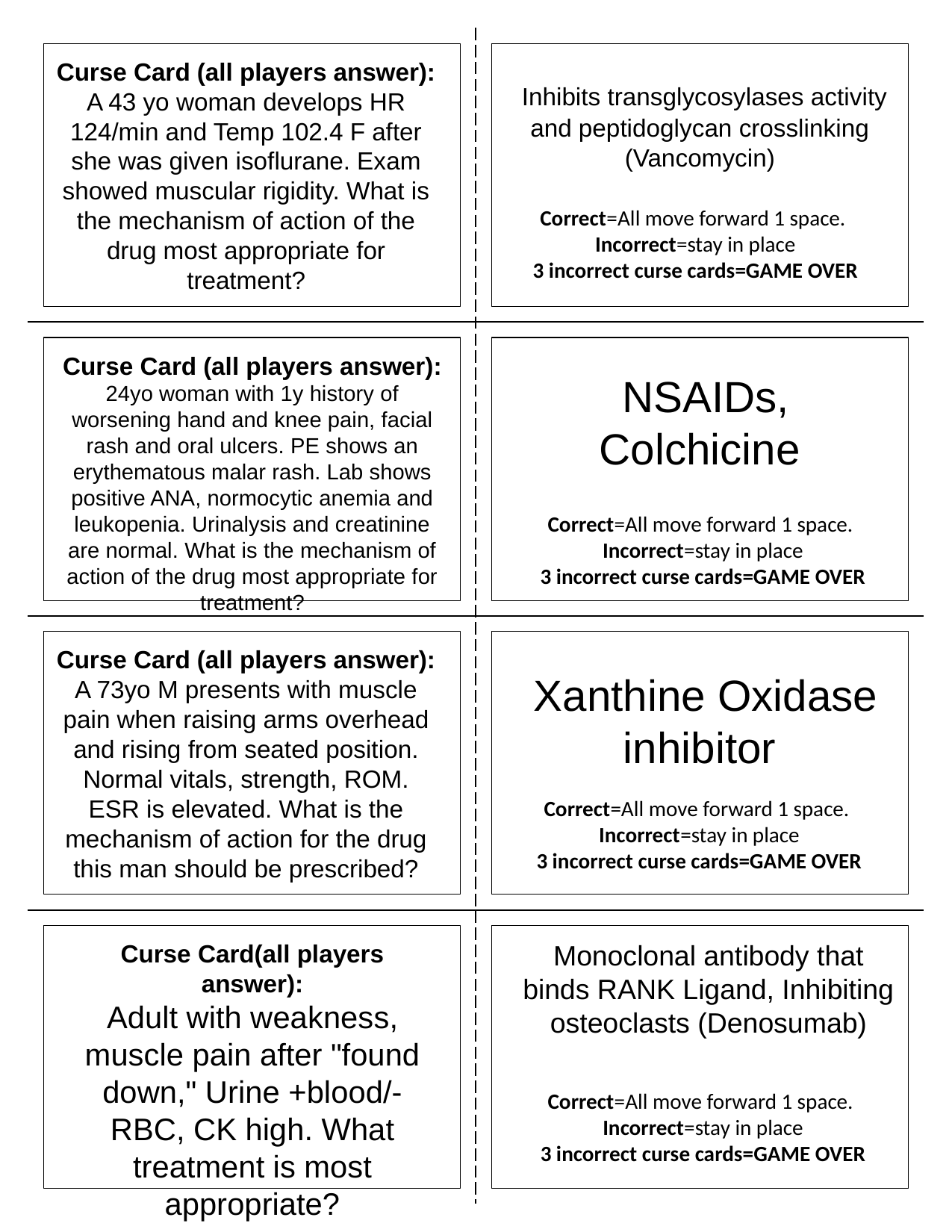

Curse Card (all players answer):
A 43 yo woman develops HR 124/min and Temp 102.4 F after she was given isoflurane. Exam showed muscular rigidity. What is the mechanism of action of the drug most appropriate for treatment?
 Inhibits transglycosylases activity and peptidoglycan crosslinking
(Vancomycin)
Correct=All move forward 1 space.
Incorrect=stay in place
3 incorrect curse cards=GAME OVER
Curse Card (all players answer):
24yo woman with 1y history of worsening hand and knee pain, facial rash and oral ulcers. PE shows an erythematous malar rash. Lab shows positive ANA, normocytic anemia and leukopenia. Urinalysis and creatinine are normal. What is the mechanism of action of the drug most appropriate for treatment?
 NSAIDs, Colchicine
Correct=All move forward 1 space.
Incorrect=stay in place
3 incorrect curse cards=GAME OVER
Curse Card (all players answer):
A 73yo M presents with muscle pain when raising arms overhead and rising from seated position. Normal vitals, strength, ROM. ESR is elevated. What is the mechanism of action for the drug this man should be prescribed?
 Xanthine Oxidase inhibitor
Correct=All move forward 1 space.
Incorrect=stay in place
3 incorrect curse cards=GAME OVER
Monoclonal antibody that binds RANK Ligand, Inhibiting osteoclasts (Denosumab)
Curse Card(all players answer):
Adult with weakness, muscle pain after "found down," Urine +blood/-RBC, CK high. What treatment is most appropriate?
Correct=All move forward 1 space.
Incorrect=stay in place
3 incorrect curse cards=GAME OVER

## Slide 16
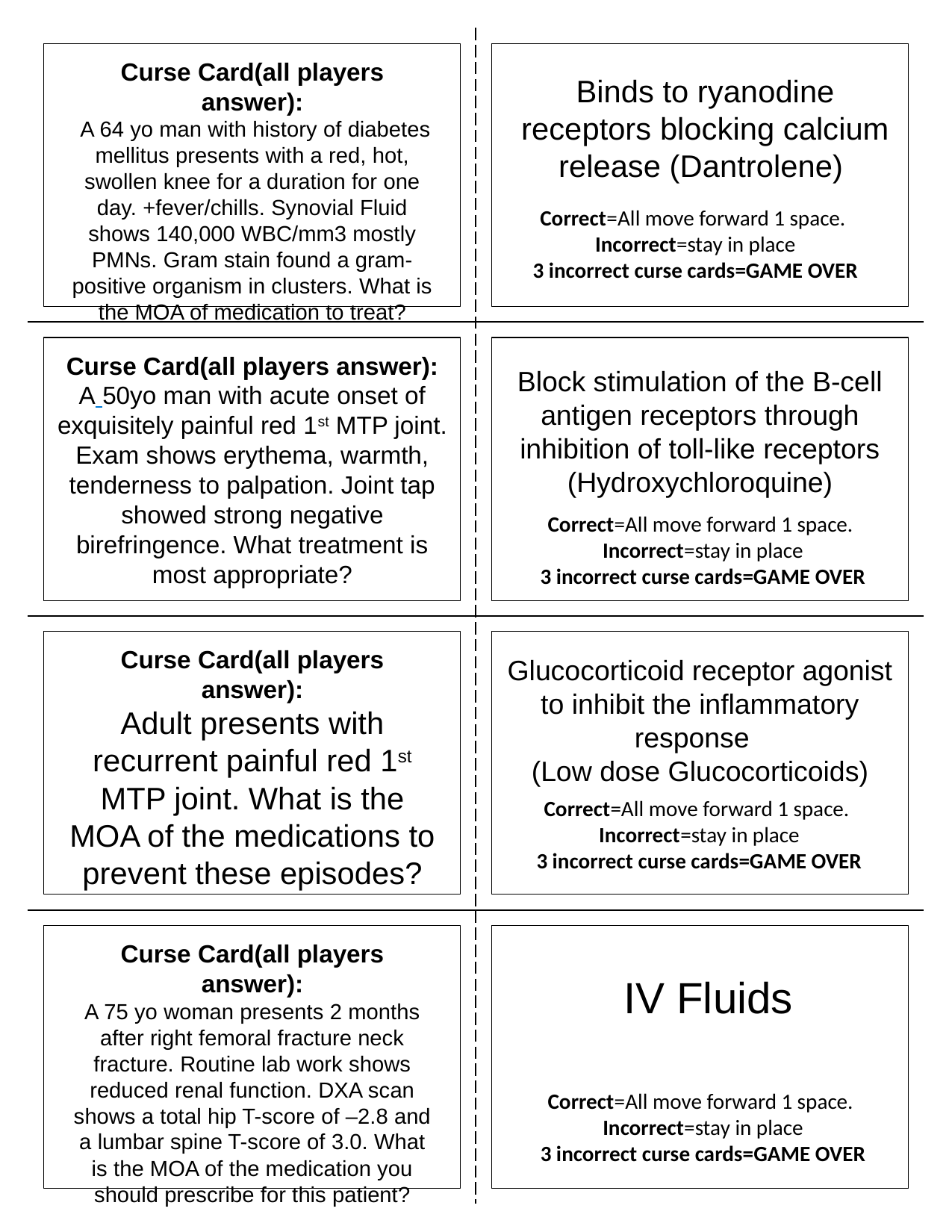

Curse Card(all players answer):
 A 64 yo man with history of diabetes mellitus presents with a red, hot, swollen knee for a duration for one day. +fever/chills. Synovial Fluid shows 140,000 WBC/mm3 mostly PMNs. Gram stain found a gram-positive organism in clusters. What is the MOA of medication to treat?
Binds to ryanodine receptors blocking calcium release (Dantrolene)
Correct=All move forward 1 space.
Incorrect=stay in place
3 incorrect curse cards=GAME OVER
Curse Card(all players answer):
A 50yo man with acute onset of exquisitely painful red 1st MTP joint. Exam shows erythema, warmth, tenderness to palpation. Joint tap showed strong negative birefringence. What treatment is most appropriate?
Block stimulation of the B-cell antigen receptors through inhibition of toll-like receptors
(Hydroxychloroquine)
Correct=All move forward 1 space.
Incorrect=stay in place
3 incorrect curse cards=GAME OVER
Curse Card(all players answer):
Adult presents with recurrent painful red 1st MTP joint. What is the MOA of the medications to prevent these episodes?
Glucocorticoid receptor agonist to inhibit the inflammatory response
(Low dose Glucocorticoids)
Correct=All move forward 1 space.
Incorrect=stay in place
3 incorrect curse cards=GAME OVER
Curse Card(all players answer):
A 75 yo woman presents 2 months after right femoral fracture neck fracture. Routine lab work shows reduced renal function. DXA scan shows a total hip T-score of –2.8 and a lumbar spine T-score of 3.0. What is the MOA of the medication you should prescribe for this patient?
IV Fluids
Correct=All move forward 1 space.
Incorrect=stay in place
3 incorrect curse cards=GAME OVER

## Slide 17
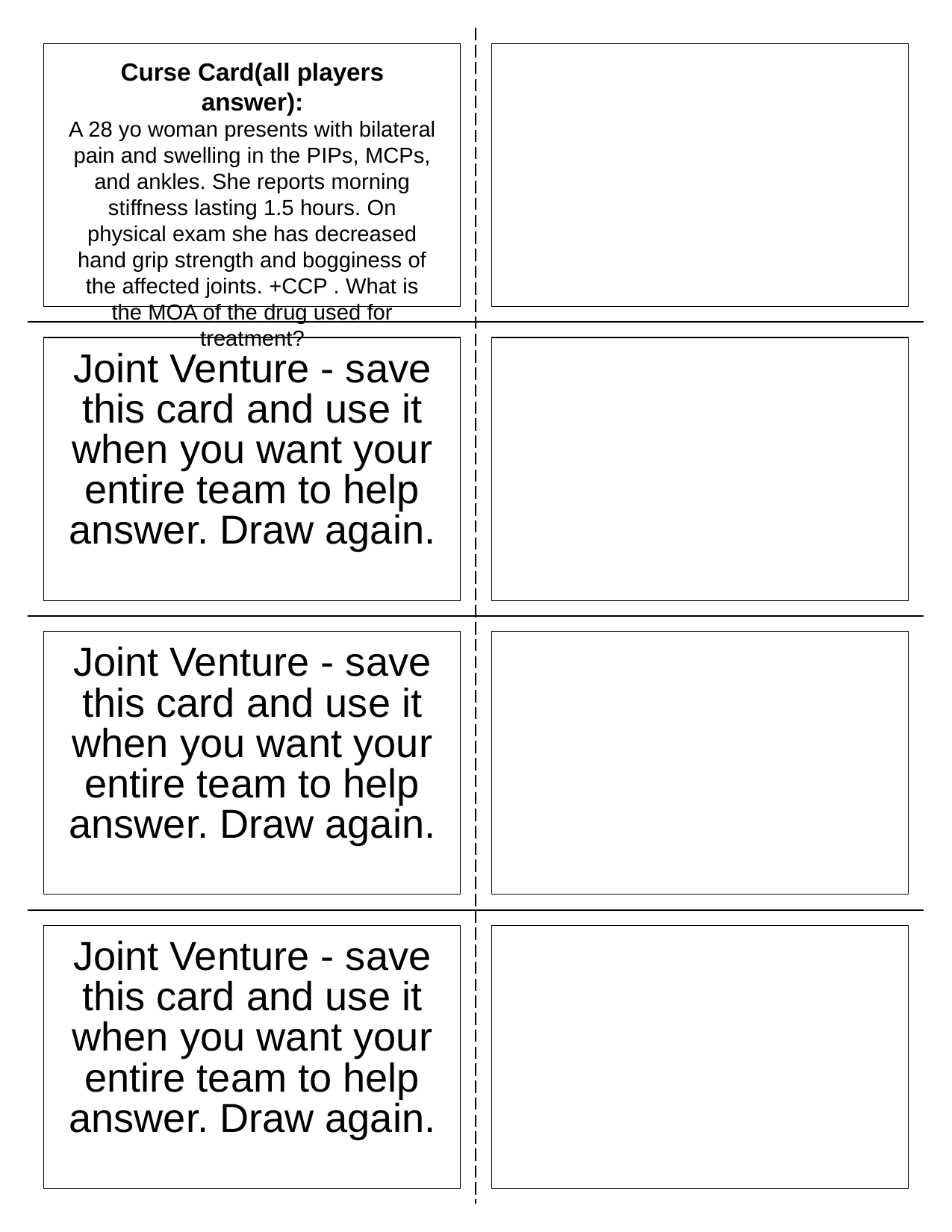

Curse Card(all players answer):
A 28 yo woman presents with bilateral pain and swelling in the PIPs, MCPs, and ankles. She reports morning stiffness lasting 1.5 hours. On physical exam she has decreased hand grip strength and bogginess of the affected joints. +CCP . What is the MOA of the drug used for treatment?
Joint Venture - save this card and use it when you want your entire team to help answer. Draw again.
Joint Venture - save this card and use it when you want your entire team to help answer. Draw again.
Joint Venture - save this card and use it when you want your entire team to help answer. Draw again.

## Slide 18
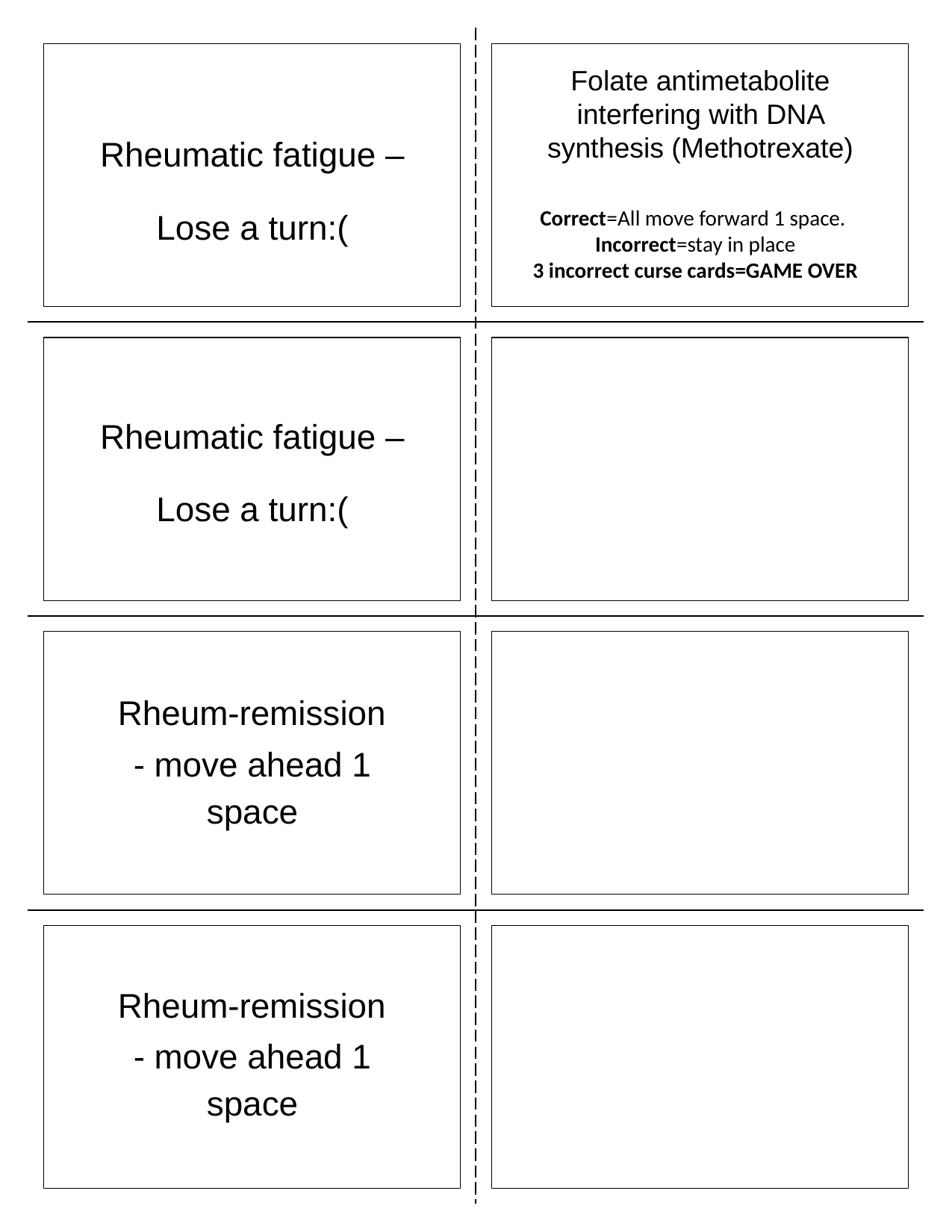

Folate antimetabolite interfering with DNA synthesis (Methotrexate)
Rheumatic fatigue – Lose a turn:(
Correct=All move forward 1 space.
Incorrect=stay in place
3 incorrect curse cards=GAME OVER
Rheumatic fatigue – Lose a turn:(
Rheum-remission
- move ahead 1 space
Rheum-remission
- move ahead 1 space

## Slide 19
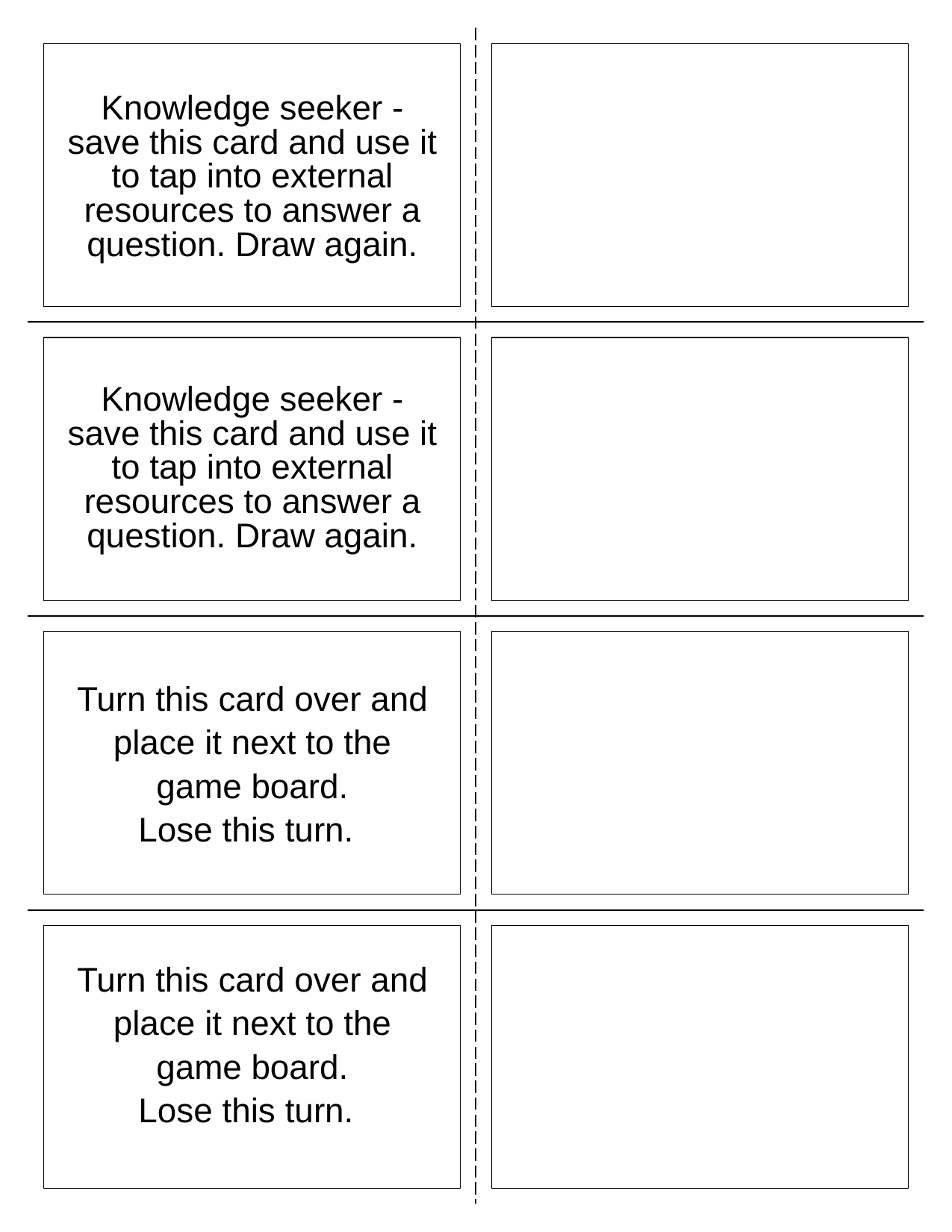

Knowledge seeker - save this card and use it to tap into external resources to answer a question. Draw again.
Knowledge seeker - save this card and use it to tap into external resources to answer a question. Draw again.
Turn this card over and place it next to the game board.
Lose this turn.
Turn this card over and place it next to the game board.
Lose this turn.
