# Supplementary material for: RheumQuest: A Gamified Approach to Musculoskeletal Education
Source: MedEdPORTAL. 2026 Mar 25;22:11587. doi: 10.15766/mep_2374-8265.11587 (PMC13013083; doi:10.15766/mep_2374-8265.11587)
Supplement: Supplementary file 1 — RheumQuest Board.pdfRheumQuest Cards.pptxRheumQuest Instructions.docxFacilitator Guide.docxPre- and Posttest with Answer Key.docx [file mep_2374-8265.11587-s001.zip › C. RheumQuest Instructions.docx]

**Appendix C: RheumQuest – Learner Instructions**

Welcome to the enchanting world of **Rheumatia**, a kingdom plagued by a malevolent curse cast by the notorious **Sorcerer Spondylosis**. As the curse takes hold, the once thriving population falls victim to mysterious and debilitating ailments, threatening the very fabric of the kingdom.

In this dire time, the noble **Lord Lupus** and the valiant **Lady Synovia** have called upon you, brave medical adventurers, to join their quest. Armed with your knowledge, you must step forward and undertake the formidable task of diagnosing and healing the kingdom’s afflicted residents before the curse engulfs them completely.

Your journey begins now. **Shuffle the card deck and place them presentation side up.** Take turns drawing cards from the deck—each card presents a clinical scenario designed to test your expertise in the realm of musculoskeletal mysteries. With each correct answer, you will follow the card’s directions and navigate through the kingdom, moving closer to uncovering the cure and dispelling the dark enchantment that plagues Rheumatia.

But beware, for the curse is relentless. The Sorcerer Spondylosis has woven dark magic into the deck itself. Nine **Curse Cards** lie hidden within the deck. If your group draws a Curse Card, all players must attempt to overcome the challenge together. Failure to answer three Curse Cards correctly will doom your fellowship and allow the curse to consume Rheumatia.

Your mission is twofold: advance across the kingdom by correctly diagnosing each presentation, and avoid falling victim to the curse’s deadly grasp. You will only succeed if **all** players reach the end of the quest. Unity, strategy, and your dedication to the healing arts are paramount.

**Instructions**

1. **Prepare the materials**: Ensure you have the game board and card deck assembled. Each player selects a pawn and places it on the start square.
2. **Determine the starting player**: The shortest player begins the adventure. Play proceeds clockwise.
3. **Gameplay**:
   - On your turn, draw the top card from the deck (presentation side up).
   - Read the clinical scenario aloud.
   - Work as a team to select the correct diagnosis or answer.
   - If correct, follow movement instructions on the card.
   - If incorrect, the student loses their turn and does not move forward.
4. **Winning the game**:
   Reach the final square with **all** players before accumulating three failed Curse Cards.
5. **Losing the game**:
   Accumulate three failed Curse Cards—the curse overwhelms the kingdom, and Rheumatia is lost.

You are now ready to begin your quest. Draw your first presentation and venture forth until you either discover the cure or fall victim to the terrible curse.

May your journey be filled with discovery, triumph, and the victory of compassion over adversity.
