# Supplementary material for: RheumQuest: A Gamified Approach to Musculoskeletal Education
Source: MedEdPORTAL. 2026 Mar 25;22:11587. doi: 10.15766/mep_2374-8265.11587 (PMC13013083; doi:10.15766/mep_2374-8265.11587)
Supplement: Supplementary file 1 — RheumQuest Board.pdfRheumQuest Cards.pptxRheumQuest Instructions.docxFacilitator Guide.docxPre- and Posttest with Answer Key.docx [file mep_2374-8265.11587-s001.zip › D. Facilitator Guide.docx]

**Appendix D: RheumQuest – Facilitator Guide**

**Purpose:**
This guide supports facilitators in preparing, conducting, and debriefing the RheumQuest musculoskeletal diagnostic reasoning game.

**Preparation**

- This activity requires approximately **60 minutes**.
- Print **game boards** and **card decks** (Appendix A & B) in color.
- Cards must be printed **double-sided**.
  **The diagnosis is printed on the reverse side, opposite the clinical presentation.**
- Cut out the cards along the guidelines and assemble them into a deck.
- Cut out player pawns and fold the bases so they stand upright.
- Print one copy of the **Learner Instructions** (Appendix C) for reference.
- Print the **pre- and post-tests** (Appendix D). Ensure half the group receives Green and half receives Yellow versions.

**Game Setup**

1. Place the complete game board on a table.
2. Place all player pawns on the **Start** square.
3. Shuffle the deck thoroughly and place it with the **clinical presentation side up**.
4. Ensure each group of three students has:
   - 1 game board
   - 1 shuffled deck
   - 3 pawns

**Gameplay Sequence**

1. The shortest player begins. Play proceeds **clockwise**.
2. On each turn:
   - The student draws the top card with the **clinical presentation side up**.
   - The group discusses and selects their answer.
   - The student flips the card to check the **diagnosis**.
3. If correct:
   - The student follows movement instructions on the card.
4. If incorrect:
   - The student **loses their turn** and does not move forward.
5. **Curse Cards:**
   - If the card is a Curse Card, all students must attempt the presentation.
   - The group records a failed curse if they answer incorrectly. Three failed curses end the game.

**Winning:**
All players reach the final square before accumulating three failed curses.

**Ending early / incomplete deck:**
If groups cannot complete the full deck within the time limit (typical), the game concludes at the 40–45-minute mark. Knowledge objectives are still met even without reaching the end.

**Facilitation Timeline**

| **Time** | **Activity** | **Notes** |
| --- | --- | --- |
| 0–2 min | Introductions & setup | Ensure materials are distributed. |
| 2–12 min | Pre-test | 10 minutes recommended. |
| 12–17 min | Explain rules & read Appendix C aloud | Clarify gameplay sequence. |
| 17–55 min | Gameplay | ~40 minutes is typical. All groups should have plenty of time to finish during this time. |
| 55–65 min | Post-test | 10 minutes. |

**Facilitator Role During Gameplay**

- Circulate among groups to answer rules questions.
- Encourage students to discuss the differential diagnosis aloud.
- Prompt higher-order reasoning if students jump straight to answers.
- Offer clarification on musculoskeletal pathophysiology when needed.

**Session Conclusion**

- Collect pre/post-tests.
- Review learner feedback.
- Re-emphasize key MSK diagnostic reasoning principles.

**Materials (per group of 2–4):**

- 1 printed game board (single-sided)
- 1 deck of double-sided cards (print double-sided; the diagnosis is printed on the reverse side opposite the clinical presentation)
- 3–6 pawns (cut and folded)
- Copies of learner instructions (Appendix C)
- Pre/post-tests (Appendix D — Green/Yellow)

**Printing note:** Print cards double-sided (flip on short edge) so each printed card contains a presentation matched with its answer on the reverse. PDF layout may show reverse sides adjacent to other cards for printing efficiency; when printed and cut each card is a matched pair.

**Setup:**

1. Assemble board on flat surface.
2. Shuffle deck thoroughly and place with clinical presentation side up.
3. Place pawns on Start.

**Session flow (recommended 60 minutes):**

- 0–10 min — Pre-test (10 minutes)
- 10–15 min — Explain rules & distribute materials
- 15–55 min — Gameplay (~35-40 minutes). This time should not be a hinderance as all groups completed the entire deck in this period. If session time is insufficient, can easily be mitigated by gauging deck size to the allotted time each educational session has.
- 55–65 min — Post-test (10 minutes)

**Gameplay sequence (per turn):**

1. Student draws the top card presentation-side up and reads the clinical presentation aloud.
2. The team discusses and agrees on an answer.
3. The student flips the card to view the correct diagnosis and brief explanation.
4. If correct, follow movement instructions on the card; if incorrect, the player’s turn ends. Curse Cards require whole-group responses; three failed curse cards end the game and you must restart from the beginning.

**Facilitation tips:**

- Encourage learners to verbalize reasoning before flipping cards.
- Keep discussions focused and limit long tangents that hinder pacing.
- If groups finish early, have them either continue drawing cards or restart the game for additional practice.
- Emphasize the formative nature of the session.

**If time runs out:** While unlikely, in the event groups need more time to complete the session. End gameplay at scheduled stop time. Consider a 5–10-minute facilitator-led review of high-yield cards or schedule a short follow-up session for remaining cards.
