# Supplementary material for: RheumQuest: A Gamified Approach to Musculoskeletal Education
Source: MedEdPORTAL. 2026 Mar 25;22:11587. doi: 10.15766/mep_2374-8265.11587 (PMC13013083; doi:10.15766/mep_2374-8265.11587)
Supplement: Supplementary file 1 — RheumQuest Board.pdfRheumQuest Cards.pptxRheumQuest Instructions.docxFacilitator Guide.docxPre- and Posttest with Answer Key.docx [file mep_2374-8265.11587-s001.zip › E. Pre- and Posttest with Answer Key.docx]

Yellow test

Pre-test answers

1. ___
2. ___
3. ___
4. ___
5. ___
6. ___
7. ___
8. ___
9. ___
10. ___
11. ___

Post-test answers

1. ___
2. ___
3. ___
4. ___
5. ___
6. ___
7. ___
8. ___
9. ___
10. ___
11. ___
12. ___
13. ___
14. ___

Yellow pre-test

1. How confident do you feel in answering questions about musculoskeletal system diagnosis?
2. not at all confident
3. slightly confident
4. moderately confident
5. quite confident
6. extremely confident
7. A 67-year-old woman presents for follow up of a right hip fracture suffered 2 months ago after a fall from standing. She denies bone and joint pain. Physical exam does not show any joint or spine deformities. Her DXA results shows osteopenia. Which of the following is the most likely diagnosis in this patient?
   1. Osteopenia
   2. Osteoporosis
   3. Osteomalacia
   4. Paget's disease
8. A 27-year-old man presents with worsening muscle weakness for the last three years and blurry vision for about 2 months. He has a medical history significant for diabetes mellitus controlled on medication. The patient reports that his mother developed similar symptoms after the age of 30. Physical exam shows normal vital signs, hair loss over the frontal scalp, and temporal muscle wasting. He has muscle weakness in the distal upper and lower extremities, but not in other areas. Which of the following is the most likely diagnosis?
   1. Becker muscular dystrophy
   2. Duchenne muscular dystrophy
   3. Ehlers Danlos classic type
   4. Marfan syndrome
   5. Myotonic dystrophy
9. A 22-year-old man presents with right heel pain for one month. The pain is worse in the morning, especially when getting out of bed, and then diminishes after walking around for 15 minutes but never goes away completely. It is partially relieved with over the counter anti-inflammatories. About 2 months ago he started running 2-3 miles every day for exercise but reports no known injury or trauma to the area. His vitals are normal. Examination shows tenderness to palpation on bottom of right foot while dorsiflexed. Which of the following is the most likely diagnosis in the patient?
   1. Achilles tendonitis
   2. Osteoarthritis
   3. Osteomyelitis
   4. Plantar fasciitis
   5. Tarsal tunnel syndrome
10. A 75-year-old man presents with right hip pain for the last 7 months. He denies any injury and has no significant past medical history. Right hip x-ray shows a lytic lesion involving the right femoral head and neck. Tests show markedly elevated serum alkaline phosphatase.
    1. Metastatic disease
    2. Osteomalacia
    3. Osteomyelitis
    4. Osteoporosis
    5. Pagets disease
11. A 25-year-old woman presents with a 2-year history of gradually worsening low back aching pain and a 1-week history of left ankle pain.  She describes worsening stiffness in the morning, often taking her almost an hour to feel like she has full mobility.  Her pain has been waking her in the middle of the night two to three times weekly.  On physical exam, she has point tenderness between the iliac crests and her spine bilaterally, worse with flexion, abduction and external rotation of the hip.  She has tenderness of the left posterior calcaneus where it meets the Achilles tendon.
    1. Ankylosing spondylitis
    2. Psoriatic arthritis
    3. Reactive arthritis
    4. Rheumatoid arthritis
    5. Spinal stenosis
12. A 37-year-old woman presents with a 10-week history of knee pain which is localized to the anterior knee.  The pain is worse after going up and down stairs or sitting for prolonged periods of time.  Exam shows no palpable swelling or effusion and range of motion of the knee is normal.  There is tenderness to palpation over the patella and pain when moving the patella medially and laterally.
    1. ACL tear
    2. Osgood-schlatter
    3. Osteoarthritis
    4. Patellofemoral pain syndrome
    5. Prepatellar bursitis
13. A 62-year-old woman presents with a 6-month history of hip and shoulder pain.  She has stiffness every morning when she wakes up, lasting up to 45 minutes until she feels loose.  On physical exam, she has normal range of motion and no tenderness.  Her strength is 5/5 in all areas.  Laboratory evaluation reveals an erythrocyte sedimentation rate of 65 mm/hour (normal <30).
    1. Fibromyalgia
    2. Inclusion body myositis
    3. Osteoarthritis
    4. Polymyalgia rheumatica
    5. Polymyositis
14. A 72-year-old woman presents with a 2-day history of worsening pain and swelling in her left elbow. She reports having 3-4 prior episodes over the past 4 years, typically affecting her knees and elbows. She describes episodes of these joints becoming swollen, red and painful, improving with ibuprofen, and resolving after 10 days. On physical exam, she is afebrile. Her left elbow is erythematous with a palpable joint effusion. She has pain throughout the motion arc but has full range of motion.
    1. Gout
    2. Pseudogout
    3. Rheumatoid arthritis
    4. Septic arthritis
    5. Transient synovitis
15. A 44-old-woman presents with wrist and finger pain over the past 6-months. She reports stiffness about 90 minutes after she wakes up in the morning. Vital signs are within normal limits. Exam shows swelling and tenderness of the wrists and metacarpophalangeal joints bilaterally. Range of motion is decreased due to pain. She has subcutaneous, nontender, firm, mobile nodules on the extensor surface of the forearm, with the overlying skin appearing normal. Which of the following is the most likely diagnosis?
    1. Gout
    2. Pseudogout
    3. Rheumatoid arthritis
    4. Sarcoidosis
    5. Systemic lupus erythematosus
16. A 43-year-old man presents with worsening right knee pain for 6 weeks. There was no known injury. He works as a mechanic. On exam, vitals are normal. There is swelling and erythema with fluctuance overlying the inferior patella but no joint line tenderness or warmth. Range of motion is limited due to pain. Which of the following is the most likely diagnosis?
    1. Gout
    2. Osteoarthritis
    3. Patellar tendonitis
    4. Prepatellar bursitis
    5. Septic arthritis

Yellow post-test

1. A 78-year-old man presents with joint pain, worse in the spine, pelvis, and legs. He has a history of celiac disease and now lives in a nursing home where he spends most of his time indoors. On exam, vitals are normal. He has difficulty bearing weight and limps when walking. He has discomfort on palpation over the anterior tibia. Which of the following is the most likely diagnosis?
2. Fibromyalgia
3. Osteomalacia
4. Osteoporosis
5. Paget’s disease
6. Rheumatoid arthritis
7. A 14-year-old boy presents with intermittent pain in the left leg, which is notably worse at night, for 2 months. The pain is relieved with ibuprofen. A plain film shows periosteal new bone formation with central radiolucent nidus on the tibia. Which of the following is the most likely diagnosis in this patient?
8. Aneurysmal bone cyst
9. Giant cell tumor of bone
10. Growing pains
11. Osteochondroma
12. Osteoid osteoma
13. A 57-year-old man presents with a 1-day history of right shoulder pain after falling out of a tree.  He reports he was trimming back some tree branches when he lost his balance and fell 6 feet, breaking his fall by stretching out his right arm.  He immediately felt a sharp pain in his right shoulder.  On physical exam, he has point tenderness on the anterior aspect of his distal shoulder.  He has full range of motion, but his pain worsens with passive adduction of his right shoulder. Which of the following is the most likely diagnosis?
14. Acromioclavicular joint separation
15. Biceps rupture
16. Brachial plexopathy
17. Glenohumeral dislocation
18. Rotator cuff tear
19. A 29-year-old man presents with intermittent numbness and tingling of his right foot for the past few months that has progressively worsened. He is a long-distance runner and notes the pain is worse with running and at night. He also notes occasional burning along the heel. Exam shows normal sensation in the feet bilaterally. Percussion inferior to the medial malleolus induces pain radiating to the medial plantar surface of his foot. Simultaneous dorsiflexion and eversion of the ankle exacerbates symptoms. There is excessive wear along the inner sole of both sides of his shoes. Which of the following is the most likely diagnosis in this patient?
20. Achilles tendonitis
21. Heel spur
22. Idiopathic polyneuropathy
23. Posterior tibial tendonitis
24. Tarsal tunnel syndrome
25. A 58-year-old woman presents with right hip pain for several years but has progressively worsened in the last few months.  She reports pain and stiffness in her right hip for 20 minutes in the morning. Pain is worse with activity during the day and improves with rest.  She denies fever, recent trauma, or recent illness.  Physical exam reveals an afebrile woman in no acute distress.  Exam of the right hip demonstrates pain when logrolling and pain on internal rotation. Range of motion testing is normal. Which of the following is the most likely diagnosis?
26. Greater trochanteric bursitis
27. Occult fracture of femoral neck
28. Osteoarthritis
29. Osteonecrosis
30. Rheumatoid arthritis
31. A 17-year-old boy presents with low back pain for the past 3 days.  He is on the school wrestling team and reports carrying a heavy backpack at school. On exam, he has paraspinal tenderness over the lumbar spine. On range of motion testing he has increased pain with hyperextension. Strength and sensation are intact in the bilateral lower extremities. Which of the following is the most likely diagnosis?
32. Ankylosing spondylitis
33. Diskitis
34. Scoliosis
35. Spinal stenosis
36. Spondylolysis
37. A 38-year-old woman presents with acute left knee pain after she cut sharply to intercept the ball during a soccer game.  She was able to walk after the game, but over the past 3 days she has had worsening knee pain and swelling.  She feels that her knee “catches” when she pivots or turns.  She localizes the pain to the joint line of the left knee.  The pain is worse with deep squatting.  On exam, pain is present with passive extension of the knee while the tibia is externally rotated and valgus stress is applied to the knee.  Based on her history and exam findings, what is the most likely source of injury?
38. Anterior cruciate ligament
39. Lateral collateral ligament
40. Lateral meniscus
41. Medial collateral ligament
42. Medial meniscus
43. A 24-year-old woman presents with a 9-month history of subjective fevers, malaise and unintentional 10-pound weight loss, and a 3-month history of diffuse arthralgias. She has otherwise been healthy and takes no medications. On physical exam she has an erythematous rash on both cheeks. Laboratory evaluation reveals a normocytic anemia, leukopenia and an elevated erythrocyte sedimentation rate. Urinalysis reveals proteinuria and hematuria. Which of the following is the most likely diagnosis?
44. Mixed connective tissue disease
45. Polymyositis
46. Rheumatoid arthritis
47. Systemic lupus erythematosus
48. Systemic Sclerosis
49. A 27-year-old man presents with a 10-month history of back pain. The pain is worst in the morning and improves with activity. He also reports bilateral hip pain and difficulty bending forward with activity. Vitals are normal. Exam shows a limited range of spinal flexion. Flexion, abduction, and external rotation of hips bilaterally reproduces pain. Which of the following is the most likely diagnosis?
50. Ankylosing spondylitis
51. Degenerative joint disease
52. Polymyositis
53. Polymyalgia rheumatica
54. Lumbago
55. A 58-year-old woman presents with a 4 day history of worsening right knee pain and 1 day history of malaise and chills. NSAIDs are not relieving the pain. Exam reveals temperature 101.9F, pulse 100/min, blood pressure 118/78mmHg, and BMI 30. Right knee is erythematous, warm, and tender to palpation. She has a large joint effusion and limitation of passive and active range of motion. Laboratory evaluation reveals WBC 10.8, ESR 78mm/hr, CRP 9.2mg/dl. Synovial fluid shows WBC 100,000, 90% PMNs. Which of the following is the most likely diagnosis?
56. Gout
57. Pseudogout
58. Septic arthritis
59. Rheumatoid arthritis
60. Prepatellar bursitis
61. **How confident do you feel in answering questions about musculoskeletal system diagnosis?**
62. not at all confident
63. slightly confident
64. moderately confident
65. quite confident
66. extremely confident
67. **Please rate how helpful the RheumQuest game was for learning musculoskeletal system diagnosis:**
68. not helpful
69. minimally helpful
70. helpful
71. very helpful
72. **What did you like about the game?**
73. **Is there anything you would change? If so, what would it be?**

Green test

Pre-test answers

1. ___
2. ___
3. ___
4. ___
5. ___
6. ___
7. ___
8. ___
9. ___
10. ___
11. ___

Post-test answers

1. ___
2. ___
3. ___
4. ___
5. ___
6. ___
7. ___
8. ___
9. ___
10. ___
11. ___
12. ___
13. ___
14. ___

Green pre-test

1. How confident do you feel in answering questions about musculoskeletal system diagnosis?
2. not at all confident
3. slightly confident
4. moderately confident
5. quite confident
6. extremely confident
7. A 78-year-old man presents with joint pain, worse in the spine, pelvis, and legs. He has a history of celiac disease and now lives in a nursing home where he spends most of his time indoors. On exam, vitals are normal. He has difficulty bearing weight and limps when walking. He has discomfort on palpation over the anterior tibia. Which of the following is the most likely diagnosis?
8. Fibromyalgia
9. Osteomalacia
10. Osteoporosis
11. Paget’s disease
12. Rheumatoid arthritis
13. A 14-year-old boy presents with intermittent pain in the left leg, which is notably worse at night, for 2 months. The pain is relieved with ibuprofen. A plain film shows periosteal new bone formation with central radiolucent nidus on the tibia. Which of the following is the most likely diagnosis in this patient?
14. Aneurysmal bone cyst
15. Giant cell tumor of bone
16. Growing pains
17. Osteochondroma
18. Osteoid osteoma
19. A 57-year-old man presents with a 1-day history of right shoulder pain after falling out of a tree.  He reports he was trimming back some tree branches when he lost his balance and fell 6 feet, breaking his fall by stretching out his right arm.  He immediately felt a sharp pain in his right shoulder.  On physical exam, he has point tenderness on the anterior aspect of his distal shoulder.  He has full range of motion, but his pain worsens with passive adduction of his right shoulder. Which of the following is the most likely diagnosis?
20. Acromioclavicular joint separation
21. Biceps rupture
22. Brachial plexopathy
23. Glenohumeral dislocation
24. Rotator cuff tear
25. A 29-year-old man presents with intermittent numbness and tingling of his right foot for the past few months that has progressively worsened. He is a long-distance runner and notes the pain is worse with running and at night. He also notes occasional burning along the heel. Exam shows normal sensation in the feet bilaterally. Percussion inferior to the medial malleolus induces pain radiating to the medial plantar surface of his foot. Simultaneous dorsiflexion and eversion of the ankle exacerbates symptoms. There is excessive wear along the inner sole of both sides of his shoes. Which of the following is the most likely diagnosis in this patient?
26. Achilles tendonitis
27. Heel spur
28. Idiopathic polyneuropathy
29. Posterior tibial tendonitis
30. Tarsal tunnel syndrome
31. A 58-year-old woman presents with right hip pain for several years but has progressively worsened in the last few months. She reports pain and stiffness in her right hip for 20 minutes in the morning. Pain is worse with activity during the day and improves with rest. She denies fever, recent trauma, or recent illness. Physical exam reveals an afebrile woman in no acute distress. Exam of the right hip demonstrates pain when logrolling and pain on internal rotation. Range of motion testing is normal. Which of the following is the most likely diagnosis?
32. Greater trochanteric bursitis
33. Occult fracture of femoral neck
34. Osteoarthritis
35. Osteonecrosis
36. Rheumatoid arthritis
37. A 17-year-old boy presents with low back pain for the past 3 days. He is on the school wrestling team and reports carrying a heavy backpack at school. On exam, he has paraspinal tenderness over the lumbar spine. On range of motion testing he has increased pain with hyperextension. Strength and sensation are intact in the bilateral lower extremities. Which of the following is the most likely diagnosis?
38. Ankylosing spondylitis
39. Diskitis
40. Scoliosis
41. Spinal stenosis
42. Spondylolysis
43. A 38-year-old woman presents with acute left knee pain after she cut sharply to intercept the ball during a soccer game. She was able to walk after the game, but over the past 3 days she has had worsening knee pain and swelling. She feels that her knee “catches” when she pivots or turns. She localizes the pain to the joint line of the left knee. The pain is worse with deep squatting. On exam, pain is present with passive extension of the knee while the tibia is externally rotated and valgus stress is applied to the knee. Based on her history and exam findings, what is the most likely source of injury?
44. Anterior cruciate ligament
45. Lateral collateral ligament
46. Lateral meniscus
47. Medial collateral ligament
48. Medial meniscus
49. A 24-year-old woman presents with a 9-month history of subjective fevers, malaise and unintentional 10-pound weight loss, and a 3-month history of diffuse arthralgias. She has otherwise been healthy and takes no medications. On physical exam she has an erythematous rash on both cheeks. Laboratory evaluation reveals a normocytic anemia, leukopenia and an elevated erythrocyte sedimentation rate. Urinalysis reveals proteinuria and hematuria. Which of the following is the most likely diagnosis?
50. Mixed connective tissue disease
51. Polymyositis
52. Rheumatoid arthritis
53. Systemic lupus erythematosus
54. Systemic Sclerosis
55. A 27-year-old man presents with a 10-month history of back pain. The pain is worst in the morning and improves with activity. He also reports bilateral hip pain and difficulty bending forward with activity. Vitals are normal. Exam shows a limited range of spinal flexion. Flexion, abduction, and external rotation of hips bilaterally reproduces pain. Which of the following is the most likely diagnosis?
56. Ankylosing spondylitis
57. Degenerative joint disease
58. Polymyositis
59. Polymyalgia rheumatica
60. Lumbago
61. A 58-year-old woman presents with a 4 day history of worsening right knee pain and 1 day history of malaise and chills. NSAIDs are not relieving the pain. Exam reveals temperature 101.9F, pulse 100/min, blood pressure 118/78mmHg, and BMI 30. Right knee is erythematous, warm, and tender to palpation. She has a large joint effusion and limitation of passive and active range of motion. Laboratory evaluation reveals WBC 10.8, ESR 78mm/hr, CRP 9.2mg/dl. Synovial fluid shows WBC 100,000, 90% PMNs. Which of the following is the most likely diagnosis?
62. Gout
63. Pseudogout
64. Septic arthritis
65. Rheumatoid arthritis
66. Prepatellar bursitis

Green post-test

1. A 67-year-old woman presents for follow up of a right hip fracture suffered 2 months ago after a fall from standing. She denies bone and joint pain. Physical exam does not show any joint or spine deformities. Her DXA results shows osteopenia. Which of the following is the most likely diagnosis in this patient?
   1. Osteopenia
   2. Osteoporosis
   3. Osteomalacia
   4. Paget's disease
2. A 27-year-old man presents with worsening muscle weakness for the last three years and blurry vision for about 2 months. He has a medical history significant for diabetes mellitus controlled on medication. The patient reports that his mother developed similar symptoms after the age of 30. Physical exam shows normal vital signs, hair loss over the frontal scalp, and temporal muscle wasting. He has muscle weakness in the distal upper and lower extremities, but not in other areas. Which of the following is the most likely diagnosis?
   1. Becker muscular dystrophy
   2. Duchenne muscular dystrophy
   3. Ehlers Danlos classic type
   4. Marfan syndrome
   5. Myotonic dystrophy
3. A 22-year-old man presents with right heel pain for one month. The pain is worse in the morning, especially when getting out of bed, and then diminishes after walking around for 15 minutes but never goes away completely. It is partially relieved with over the counter anti-inflammatories. About 2 months ago he started running 2-3 miles every day for exercise but reports no known injury or trauma to the area. His vitals are normal. Examination shows tenderness to palpation on bottom of right foot while dorsiflexed. Which of the following is the most likely diagnosis in the patient?
   1. Achilles tendonitis
   2. Osteoarthritis
   3. Osteomyelitis
   4. Plantar fasciitis
   5. Tarsal tunnel syndrome
4. A 75-year-old man presents with right hip pain for the last 7 months. He denies any injury and has no significant past medical history. Right hip x-ray shows a lytic lesion involving the right femoral head and neck. Tests show markedly elevated serum alkaline phosphatase.
   1. Metastatic disease
   2. Osteomalacia
   3. Osteomyelitis
   4. Osteoporosis
   5. Pagets disease
5. A 25-year-old woman presents with a 2-year history of gradually worsening low back aching pain and a 1-week history of left ankle pain. She describes worsening stiffness in the morning, often taking her almost an hour to feel like she has full mobility. Her pain has been waking her in the middle of the night two to three times weekly. On physical exam, she has point tenderness between the iliac crests and her spine bilaterally, worse with flexion, abduction and external rotation of the hip. She has tenderness of the left posterior calcaneus where it meets the Achilles tendon.
   1. Ankylosing spondylitis
   2. Psoriatic arthritis
   3. Reactive arthritis
   4. Rheumatoid arthritis
   5. Spinal stenosis
6. A 37-year-old woman presents with a 10-week history of knee pain which is localized to the anterior knee. The pain is worse after going up and down stairs or sitting for prolonged periods of time. Exam shows no palpable swelling or effusion and range of motion of the knee is normal. There is tenderness to palpation over the patella and pain when moving the patella medially and laterally.
   1. ACL tear
   2. Osgood-schlatter
   3. Osteoarthritis
   4. Patellofemoral pain syndrome
   5. Prepatellar bursitis
7. A 62-year-old woman presents with a 6-month history of hip and shoulder pain. She has stiffness every morning when she wakes up, lasting up to 45 minutes until she feels loose. On physical exam, she has normal range of motion and no tenderness. Her strength is 5/5 in all areas. Laboratory evaluation reveals an erythrocyte sedimentation rate of 65 mm/hour (normal <30).
   1. Fibromyalgia
   2. Inclusion body myositis
   3. Osteoarthritis
   4. Polymyalgia rheumatica
   5. Polymyositis
8. A 72-year-old woman presents with a 2-day history of worsening pain and swelling in her left elbow. She reports having 3-4 prior episodes over the past 4 years, typically affecting her knees and elbows. She describes episodes of these joints becoming swollen, red and painful, improving with ibuprofen, and resolving after 10 days. On physical exam, she is afebrile. Her left elbow is erythematous with a palpable joint effusion. She has pain throughout the motion arc but has full range of motion.
   1. Gout
   2. Pseudogout
   3. Rheumatoid arthritis
   4. Septic arthritis
   5. Transient synovitis
9. A 44-old-woman presents with wrist and finger pain over the past 6-months. She reports stiffness about 90 minutes after she wakes up in the morning. Vital signs are within normal limits. Exam shows swelling and tenderness of the writsts and metacarpophalangeal joints bilaterally. Range of motion is decreased due to pain. She has subcutaneous, nontender, firm, mobile nodules on the extensor surface of the forearm, with the overlying skin appearing normal. Which of the following is the most likely diagnosis?
   1. Gout
   2. Pseudogout
   3. Rheumatoid arthritis
   4. Sarcoidosis
   5. Systemic lupus erythematosis
10. A 43-year-old man presents with worsening right knee pain for 6 weeks. There was no known injury. He works as a mechanic. On exam, vitals are normal. There is swelling and erythema with fluctuance overlying the inferior patella but no joint line tenderness or warmth. Range of motion is limited due to pain. Which of the following is the most likely diagnosis?
    1. Gout
    2. Osteoarthritis
    3. Patellar tendonitis
    4. Prepatellar bursitis
    5. Septic arthritis
11. **How confident do you feel in answering questions about musculoskeletal system diagnosis?**
12. not at all confident
13. slightly confident
14. moderately confident
15. quite confident
16. extremely confident
17. **Please rate how helpful the RheumQuest game was for learning musculoskeletal system diagnosis:**
18. not helpful
19. minimally helpful
20. helpful
21. very helpful
22. **What did you like about the game?**
23. **Is there anything you would change? If so, what would it be?**

| **Key** | | | |
| --- | --- | --- | --- |
| **Yellow** | | **Green** | |
| **Pre** | **Post** | **Pre** | **Post** |
| 1) Student Directed | 1) B | 1) Student Directed | 1) B |
| 2) B | 2) E | 2) B | 2) E |
| 3) E | 3) A | 3) E | 3) D |
| 4) D | 4) E | 4) A | 4) E |
| 5) E | 5) C | 5) E | 5) A |
| 6) A | 6) E | 6) C | 6) D |
| 7) D | 7) E | 7) E | 7) D |
| 8) D | 8) D | 8) E | 8) B |
| 9) B | 9) A | 9) D | 9) C |
| 10) C | 10) C | 10) A | 10) D |
| 11) D |  | 11) C |  |

**Yellow Pre-Test**

2. Osteoporosis after fragility fracture

Correct answer: b. Osteoporosis

Why correct:

A hip fracture from a fall from standing height is a *fragility fracture* — this automatically establishes a diagnosis of osteoporosis, regardless of DXA score.
Even though her DXA shows osteopenia, the presence of a fragility fracture supersedes the T-score.

Why the others are wrong:

- a. Osteopenia — Incorrect; osteopenia by DXA alone cannot be diagnosed *after* a fragility fracture.
- c. Osteomalacia — Would present with bone pain, muscle weakness, and abnormal labs (↓Ca, ↓PO₄, ↑ALP). She has none.
- d. Paget’s disease — Usually shows bony deformity, focal enlargement, and very elevated ALP; not consistent with clinical picture.

3. Myotonic dystrophy

Correct answer: e. Myotonic dystrophy

Why correct:

Classic findings:

- Distal muscle weakness
- Frontal balding
- Cataracts (causing blurry vision)
- Family history (autosomal dominant)
- Temporal wasting
  These are hallmark features of myotonic dystrophy type 1.

Why the others are wrong:

- a. Becker MD — Proximal weakness, onset in adolescence; no cataracts or balding.
- b. Duchenne MD — Early childhood onset, rapid progression; patient is too old.
- c. Ehlers-Danlos — Features include hyperextensible skin and hypermobile joints, not weakness.
- d. Marfan — Tall stature, lens dislocation, aortic root dilation — none present.

4. Plantar fasciitis

Correct answer: d. Plantar fasciitis

Why correct:

Key features:

- Heel pain worst with first steps in morning
- Improved with walking
- Tenderness over plantar fascia insertion
- Associated with increased activity
  Classic presentation.

Why the others are wrong:

- a. Achilles tendonitis — Pain is posterior heel, not plantar surface.
- b. Osteoarthritis — Rare in ankle/foot at this age and not activity-pattern matched.
- c. Osteomyelitis — Would have fever, systemic symptoms, pain not improving with activity.
- e. Tarsal tunnel syndrome — Produces neuropathic symptoms (tingling, numbness), not morning start-up pain.

5. Paget’s disease of bone

Correct answer: e. Paget’s disease

Why correct:

- Lytic lesion in femoral head/neck
- Markedly ↑ alkaline phosphatase
- Older patient
  Paget’s disease shows mixed lytic/sclerotic lesions and very high ALP.

Why incorrect choices:

- a. Metastatic disease — ALP can be high, but lesions are rarely described as classic Paget pattern.
- b. Osteomalacia — ALP mildly ↑; x-rays show Looser zones.
- c. Osteomyelitis — Requires fever/WBC changes.
- d. Osteoporosis — Does *not* cause lytic lesions or ↑ALP.

6. Ankylosing spondylitis

Correct answer: a. Ankylosing spondylitis

Why correct:

- Chronic inflammatory low back pain
- Morning stiffness >30 min
- Night pain
- Sacroiliac involvement
- Enthesitis (Achilles tenderness)
  Classic AS features.

Why the others are wrong:

- b. Psoriatic arthritis — Needs skin/nail findings.
- c. Reactive arthritis — Requires recent GU/GI infection.
- d. Rheumatoid arthritis — Symmetric small joint disease; not axial.
- e. Spinal stenosis — Worse with extension, relieved with sitting; not morning stiffness.

7. Patellofemoral pain syndrome

Correct answer: d. Patellofemoral pain syndrome

Why correct:

- Anterior knee pain
- Worse with stairs & prolonged sitting (“movie-theater sign”)
- Patellar tenderness & pain with patellar grind test
  Classic PFPS presentation.

Why others are wrong:

- a. ACL tear — Acute trauma with instability.
- b. Osgood-Schlatter — Teens, tibial tubercle pain.
- c. Osteoarthritis — Crepitus, older adults.
- e. Prepatellar bursitis — Anterior swelling over kneecap.

8. Polymyalgia rheumatica

Correct answer: d. Polymyalgia rheumatica

Why correct:

- Older adult
- Bilateral shoulder/hip girdle pain
- Morning stiffness
- Normal strength
- Very elevated ESR
  Classic PMR.

Why others are wrong:

- a. Fibromyalgia — Normal labs, diffuse pain, poor sleep.
- b. IBM — Weakness, not just stiffness.
- c. OA — Pain with movement, normal ESR.
- e. Polymyositis — Weakness, ↑CK.

9. Pseudogout

Correct answer: b. Pseudogout

Why correct:

- Recurrent acute monoarthritis in older adult
- Large joints (knee, elbow)
- Erythema, swelling
  Presentation is classic for CPPD attacks.

Why others are wrong:

- a. Gout — Classically 1st MTP; elbow less common.
- c. RA — Chronic, symmetric, not episodic.
- d. Septic arthritis — Single severe episode, fever, worse systemic involvement.
- e. Transient synovitis — Children, typically hip.

10. Rheumatoid arthritis

Correct answer: c. Rheumatoid arthritis

Why correct:

- Symmetric wrist & MCP arthritis
- Morning stiffness >60 min
- Rheumatoid nodules
  Classic RA picture.

Why others are wrong:

- a. Gout — Episodic, not symmetric small joint involvement.
- b. Pseudogout — Large joints, chondrocalcinosis.
- d. Sarcoidosis — Non-caseating granulomas, pulmonary involvement.
- e. SLE — Non-erosive arthritis, systemic involvement.

11. Prepatellar bursitis

Correct answer: d. Prepatellar bursitis

Why correct:

- Anterior knee swelling over the patella, not in joint space
- Tender fluctuant swelling
- Mechanic occupation → frequent kneeling
  Classic bursitis.

Why others are wrong:

- a. Gout — Episodic attacks, often MTP.
- b. OA — Chronic, not acute swelling.
- c. Patellar tendonitis — Pain at inferior pole of patella, not swelling.
- e. Septic arthritis — Joint line swelling + fevers.

**Yellow Post- Test**

1. Osteomalacia

Correct answer: b. Osteomalacia

Why correct:

- Celiac disease → vitamin D deficiency
- Bone pain with difficulty weight-bearing
- Tibial tenderness
- Minimal sun exposure
  Classic osteomalacia.

Why others are wrong:

- a. Fibromyalgia — Widespread pain but no bone tenderness.
- c. Osteoporosis — Fragility fractures but not bone pain.
- d. Paget’s — Very high ALP, bony deformities.
- e. RA — Polyarticular joint inflammation.

2. Osteoid osteoma

Correct answer: e. Osteoid osteoma

Why correct:

- Night pain
- Relieved by NSAIDs
- X-ray: radiolucent nidus with reactive sclerosis
  Classic osteoid osteoma.

Why others are wrong:

- a. ABC — Expansile, blood-filled lesion.
- b. Giant cell tumor — Epiphyseal × young adults.
- c. Growing pains — No imaging abnormalities.
- d. Osteochondroma — Bony stalk, not nidus.

3. Acromioclavicular joint separation

Correct answer: a. AC joint separation

Why correct:

- Fall onto outstretched arm
- Pain localized to AC joint
- Pain with cross-body adduction
  Classic “shoulder separation.”

Why others are wrong:

- b. Biceps rupture — Popeye deformity.
- c. Brachial plexopathy — Neuro deficits.
- d. GH dislocation — Loss of ROM, deformity.
- e. Rotator cuff tear — Weakness with abduction.

4. Tarsal tunnel syndrome

Correct answer: e. Tarsal tunnel syndrome

Why correct:

- Burning/tingling medial plantar foot
- Worse with running
- Positive Tinel sign inferior to medial malleolus
- Symptoms with dorsiflexion/eversion
  Classic tibial nerve entrapment.

Why others are wrong:

- a. Achilles tendonitis — Posterior heel pain.
- b. Heel spur — Localized heel pain, no neurology.
- c. Polyneuropathy — Bilateral, stocking-glove.
- d. Tibial tendonitis — Medial ankle pain, not neuropathic symptoms.

5. Osteoarthritis

Correct answer: c. Osteoarthritis

Why correct:

- Gradual hip pain in older adult
- Morning stiffness <30 min
- Worse with activity, relieved with rest
- Pain with logroll/internal rotation
  Classic OA.

Why others are wrong:

- a. GT bursitis — Lateral hip pain.
- b. Occult fracture — Acute onset, severe pain.
- d. Osteonecrosis — Risk factors absent; severe pain.
- e. RA — Symmetric polyarthritis.

6. Spondylolysis

Correct answer: e. Spondylolysis

Why correct:

- Adolescent athlete
- Low back pain worse with extension
- Paraspinal tenderness
  Classic pars interarticularis stress fracture.

Why others are wrong:

- a. AS — Chronic inflammatory stiffness, not acute.
- b. Diskitis — Fever, systemic symptoms.
- c. Scoliosis — Asymmetrical spine, not acute pain.
- d. Stenosis — Older adults.

7. Medial meniscus tear

Correct answer: e. Medial meniscus

Why correct:

- Sudden pivot injury
- Joint line pain
- Locking/catching
- Pain with McMurray-like test
  Classic meniscus tear.

Why others are wrong:

- a. ACL — Pop, swelling, instability.
- b. LCL — Lateral pain.
- c. Lateral meniscus — Pain on lateral joint line.
- d. MCL — Valgus injury with instability.

8. Systemic lupus erythematosus

Correct answer: d. SLE

Why correct:

- Malar rash
- Systemic symptoms (weight loss, fevers)
- Anemia, leukopenia
- Renal involvement
  Classic SLE.

Why others are wrong:

- a. MCTD — Anti-U1 RNP, overlap features.
- b. Polymyositis — Muscle weakness, ↑CK.
- c. RA — Joint symptoms without rash/renal disease.
- e. Scleroderma — Skin thickening/Raynaud disease.

9. Ankylosing spondylitis

Correct answer: a. Ankylosing spondylitis

Why correct:

- Chronic inflammatory back pain
- Improves with activity
- Reduced spinal flexion
- HIP enthesitis reproduced with FABER
  Classic AS.

Why others are wrong:

- b. DJD — Worse with activity.
- c. Polymyositis — Proximal weakness.
- d. PMR — Older patients + normal strength.
- e. Lumbago — Non-inflammatory acute pain.

10. Septic arthritis

Correct answer: c. Septic arthritis

Why correct:

- Acute monoarthritis
- Fever and chills
- Very high ESR/CRP
- Synovial WBC >50,000 with 90% PMNs
  This is textbook septic arthritis.

Why others are wrong:

- a. Gout — Needle-shaped crystals, usually lower WBC.
- b. Pseudogout — Rhomboid crystals.
- d. RA — Chronic disease, not acute hot joint.
- e. Prepatellar bursitis — Overlying bursa, not joint effusion.

**Green Pre- Test**

2. Osteomalacia

Correct answer: b. Osteomalacia

Why correct:

- Celiac disease → vitamin D deficiency
- Bone pain with difficulty weight-bearing
- Tibial tenderness
- Minimal sun exposure
  Classic osteomalacia.

Why others are wrong:

- a. Fibromyalgia — Widespread pain but no bone tenderness.
- c. Osteoporosis — Fragility fractures but not bone pain.
- d. Paget’s — Very high ALP, bony deformities.
- e. RA — Polyarticular joint inflammation.

3. Osteoid osteoma

Correct answer: e. Osteoid osteoma

Why correct:

- Night pain
- Relieved by NSAIDs
- X-ray: radiolucent nidus with reactive sclerosis
  Classic osteoid osteoma.

Why others are wrong:

- a. ABC — Expansile, blood-filled lesion.
- b. Giant cell tumor — Epiphyseal × young adults.
- c. Growing pains — No imaging abnormalities.
- d. Osteochondroma — Bony stalk, not nidus.

4. Acromioclavicular joint separation

Correct answer: a. AC joint separation

Why correct:

- Fall onto outstretched arm
- Pain localized to AC joint
- Pain with cross-body adduction
  Classic “shoulder separation.”

Why others are wrong:

- b. Biceps rupture — Popeye deformity.
- c. Brachial plexopathy — Neuro deficits.
- d. GH dislocation — Loss of ROM, deformity.
- e. Rotator cuff tear — Weakness with abduction.

5. Tarsal tunnel syndrome

Correct answer: e. Tarsal tunnel syndrome

Why correct:

- Burning/tingling medial plantar foot
- Worse with running
- Positive Tinel sign inferior to medial malleolus
- Symptoms with dorsiflexion/eversion
  Classic tibial nerve entrapment.

Why others are wrong:

- a. Achilles tendonitis — Posterior heel pain.
- b. Heel spur — Localized heel pain, no neurology.
- c. Polyneuropathy — Bilateral, stocking-glove.
- d. Tibial tendonitis — Medial ankle pain, not neuropathic symptoms.

6. Osteoarthritis

Correct answer: c. Osteoarthritis

Why correct:

- Gradual hip pain in older adult
- Morning stiffness <30 min
- Worse with activity, relieved with rest
- Pain with logroll/internal rotation
  Classic OA.

Why others are wrong:

- a. GT bursitis — Lateral hip pain.
- b. Occult fracture — Acute onset, severe pain.
- d. Osteonecrosis — Risk factors absent; severe pain.
- e. RA — Symmetric polyarthritis.

7. Spondylolysis

Correct answer: e. Spondylolysis

Why correct:

- Adolescent athlete
- Low back pain worse with extension
- Paraspinal tenderness
  Classic pars interarticularis stress fracture.

Why others are wrong:

- a. AS — Chronic inflammatory stiffness, not acute.
- b. Diskitis — Fever, systemic symptoms.
- c. Scoliosis — Asymmetrical spine, not acute pain.
- d. Stenosis — Older adults.

8. Medial meniscus tear

Correct answer: e. Medial meniscus

Why correct:

- Sudden pivot injury
- Joint line pain
- Locking/catching
- Pain with McMurray-like test
  Classic meniscus tear.

Why others are wrong:

- a. ACL — Pop, swelling, instability.
- b. LCL — Lateral pain.
- c. Lateral meniscus — Pain on lateral joint line.
- d. MCL — Valgus injury with instability.

9. Systemic lupus erythematosus

Correct answer: d. SLE

Why correct:

- Malar rash
- Systemic symptoms (weight loss, fevers)
- Anemia, leukopenia
- Renal involvement
  Classic SLE.

Why others are wrong:

- a. MCTD — Anti-U1 RNP, overlap features.
- b. Polymyositis — Muscle weakness, ↑CK.
- c. RA — Joint symptoms without rash/renal disease.
- e. Scleroderma — Skin thickening/Raynaud disease.

10. Ankylosing spondylitis

Correct answer: a. Ankylosing spondylitis

Why correct:

- Chronic inflammatory back pain
- Improves with activity
- Reduced spinal flexion
- HIP enthesitis reproduced with FABER
  Classic AS.

Why others are wrong:

- b. DJD — Worse with activity.
- c. Polymyositis — Proximal weakness.
- d. PMR — Older patients + normal strength.
- e. Lumbago — Non-inflammatory acute pain.

11. Septic arthritis

Correct answer: c. Septic arthritis

Why correct:

- Acute monoarthritis
- Fever and chills
- Very high ESR/CRP
- Synovial WBC >50,000 with 90% PMNs
  This is textbook septic arthritis.

Why others are wrong:

- a. Gout — Needle-shaped crystals, usually lower WBC.
- b. Pseudogout — Rhomboid crystals.
- d. RA — Chronic disease, not acute hot joint.
- e. Prepatellar bursitis — Overlying bursa, not joint effusion.

**Green Post- Test**

1. Osteoporosis after fragility fracture

Correct answer: b. Osteoporosis

Why correct:

A hip fracture from a fall from standing height is a *fragility fracture* — this automatically establishes a diagnosis of osteoporosis, regardless of DXA score.
Even though her DXA shows osteopenia, the presence of a fragility fracture supersedes the T-score.

Why the others are wrong:

- a. Osteopenia — Incorrect; osteopenia by DXA alone cannot be diagnosed *after* a fragility fracture.
- c. Osteomalacia — Would present with bone pain, muscle weakness, and abnormal labs (↓Ca, ↓PO₄, ↑ALP). She has none.
- d. Paget’s disease — Usually shows bony deformity, focal enlargement, and very elevated ALP; not consistent with clinical picture.

2. Myotonic dystrophy

Correct answer: e. Myotonic dystrophy

Why correct:

Classic findings:

- Distal muscle weakness
- Frontal balding
- Cataracts (causing blurry vision)
- Family history (autosomal dominant)
- Temporal wasting
  These are hallmark features of myotonic dystrophy type 1.

Why the others are wrong:

- a. Becker MD — Proximal weakness, onset in adolescence; no cataracts or balding.
- b. Duchenne MD — Early childhood onset, rapid progression; patient is too old.
- c. Ehlers-Danlos — Features include hyperextensible skin and hypermobile joints, not weakness.
- d. Marfan — Tall stature, lens dislocation, aortic root dilation — none present.

3. Plantar fasciitis

Correct answer: d. Plantar fasciitis

Why correct:

Key features:

- Heel pain worst with first steps in morning
- Improved with walking
- Tenderness over plantar fascia insertion
- Associated with increased activity
  Classic presentation.

Why the others are wrong:

- a. Achilles tendonitis — Pain is posterior heel, not plantar surface.
- b. Osteoarthritis — Rare in ankle/foot at this age and not activity-pattern matched.
- c. Osteomyelitis — Would have fever, systemic symptoms, pain not improving with activity.
- e. Tarsal tunnel syndrome — Produces neuropathic symptoms (tingling, numbness), not morning start-up pain.

4. Paget’s disease of bone

Correct answer: e. Paget’s disease

Why correct:

- Lytic lesion in femoral head/neck
- Markedly ↑ alkaline phosphatase
- Older patient
  Paget’s disease shows mixed lytic/sclerotic lesions and very high ALP.

Why incorrect choices:

- a. Metastatic disease — ALP can be high, but lesions are rarely described as classic Paget pattern.
- b. Osteomalacia — ALP mildly ↑; x-rays show Looser zones.
- c. Osteomyelitis — Requires fever/WBC changes.
- d. Osteoporosis — Does *not* cause lytic lesions or ↑ALP.

5. Ankylosing spondylitis

Correct answer: a. Ankylosing spondylitis

Why correct:

- Chronic inflammatory low back pain
- Morning stiffness >30 min
- Night pain
- Sacroiliac involvement
- Enthesitis (Achilles tenderness)
  Classic AS features.

Why the others are wrong:

- b. Psoriatic arthritis — Needs skin/nail findings.
- c. Reactive arthritis — Requires recent GU/GI infection.
- d. Rheumatoid arthritis — Symmetric small joint disease; not axial.
- e. Spinal stenosis — Worse with extension, relieved with sitting; not morning stiffness.

6. Patellofemoral pain syndrome

Correct answer: d. Patellofemoral pain syndrome

Why correct:

- Anterior knee pain
- Worse with stairs & prolonged sitting (“movie-theater sign”)
- Patellar tenderness & pain with patellar grind test
  Classic PFPS presentation.

Why others are wrong:

- a. ACL tear — Acute trauma with instability.
- b. Osgood-Schlatter — Teens, tibial tubercle pain.
- c. Osteoarthritis — Crepitus, older adults.
- e. Prepatellar bursitis — Anterior swelling over kneecap.

7. Polymyalgia rheumatica

Correct answer: d. Polymyalgia rheumatica

Why correct:

- Older adult
- Bilateral shoulder/hip girdle pain
- Morning stiffness
- Normal strength
- Very elevated ESR
  Classic PMR.

Why others are wrong:

- a. Fibromyalgia — Normal labs, diffuse pain, poor sleep.
- b. IBM — Weakness, not just stiffness.
- c. OA — Pain with movement, normal ESR.
- e. Polymyositis — Weakness, ↑CK.

8. Pseudogout

Correct answer: b. Pseudogout

Why correct:

- Recurrent acute monoarthritis in older adult
- Large joints (knee, elbow)
- Erythema, swelling
  Presentation is classic for CPPD attacks.

Why others are wrong:

- a. Gout — Classically 1st MTP; elbow less common.
- c. RA — Chronic, symmetric, not episodic.
- d. Septic arthritis — Single severe episode, fever, worse systemic involvement.
- e. Transient synovitis — Children, typically hip.

9. Rheumatoid arthritis

Correct answer: c. Rheumatoid arthritis

Why correct:

- Symmetric wrist & MCP arthritis
- Morning stiffness >60 min
- Rheumatoid nodules
  Classic RA picture.

Why others are wrong:

- a. Gout — Episodic, not symmetric small joint involvement.
- b. Pseudogout — Large joints, chondrocalcinosis.
- d. Sarcoidosis — Non-caseating granulomas, pulmonary involvement.
- e. SLE — Non-erosive arthritis, systemic involvement.

10. Prepatellar bursitis

Correct answer: d. Prepatellar bursitis

Why correct:

- Anterior knee swelling over the patella, not in joint space
- Tender fluctuant swelling
- Mechanic occupation → frequent kneeling
  Classic bursitis.

Why others are wrong:

- a. Gout — Episodic attacks, often MTP.
- b. OA — Chronic, not acute swelling.
- c. Patellar tendonitis — Pain at inferior pole of patella, not swelling.
- e. Septic arthritis — Joint line swelling + fevers.
