# Supplementary figures and images for: RheumQuest: A Gamified Approach to Musculoskeletal Education
Source: MedEdPORTAL. 2026 Mar 25;22:11587. doi: 10.15766/mep_2374-8265.11587 (PMC13013083; doi:10.15766/mep_2374-8265.11587)

All Images Author Owned.

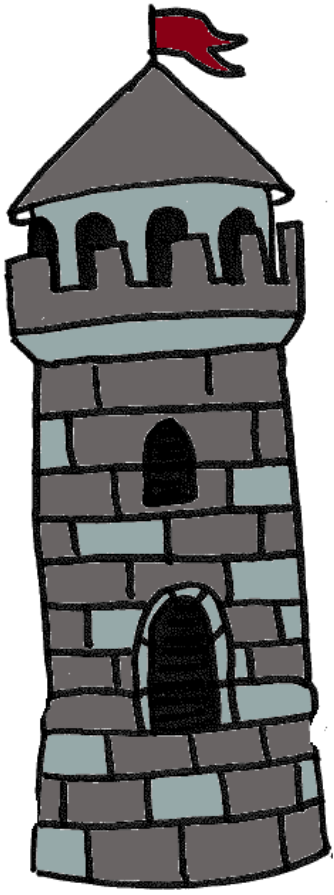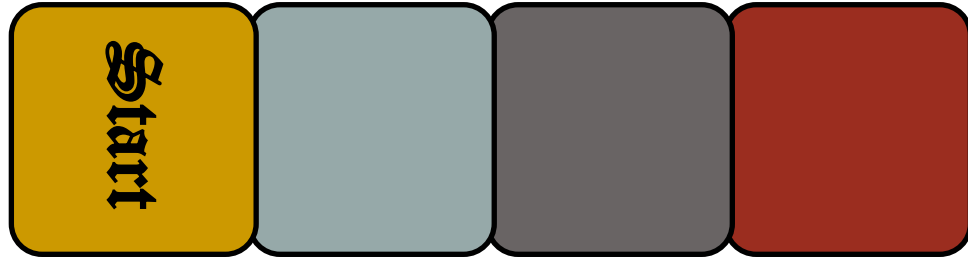

# RheumQuest

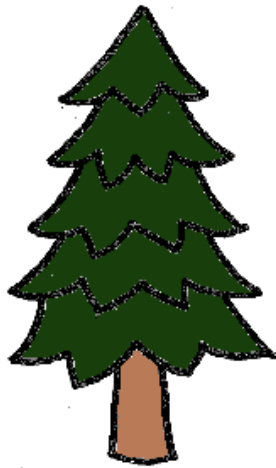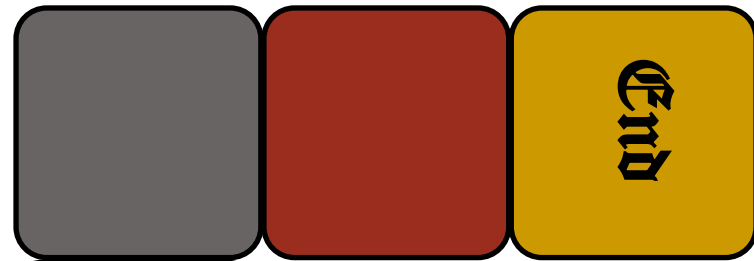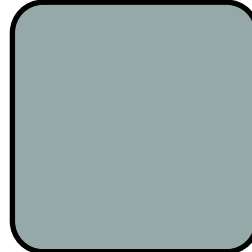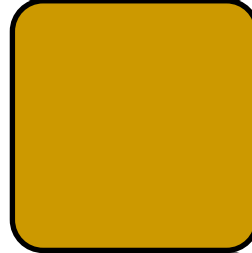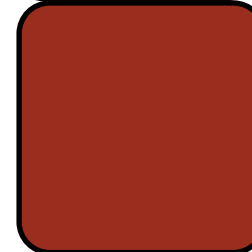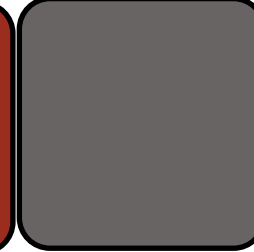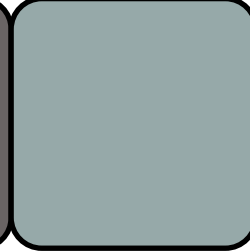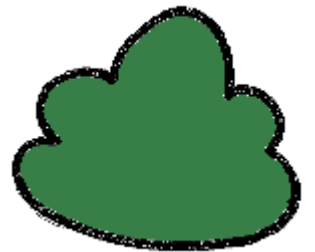

Cut

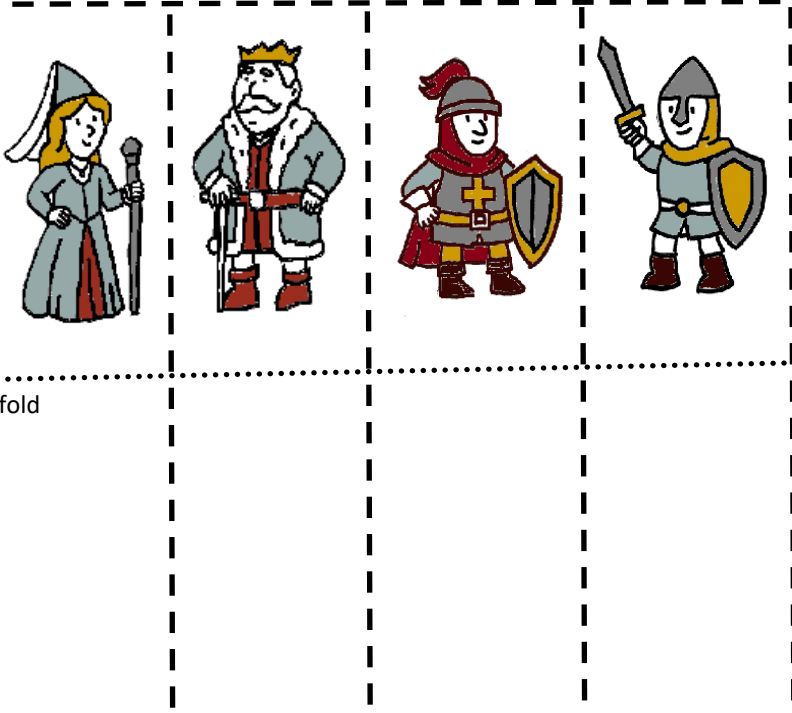

fold

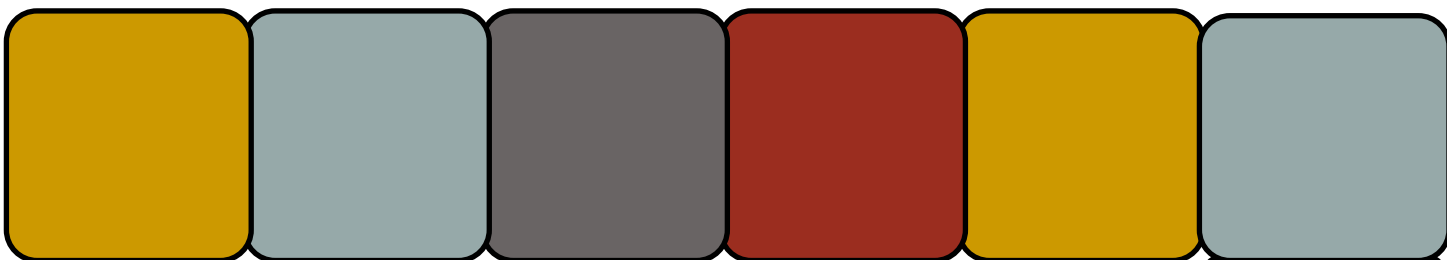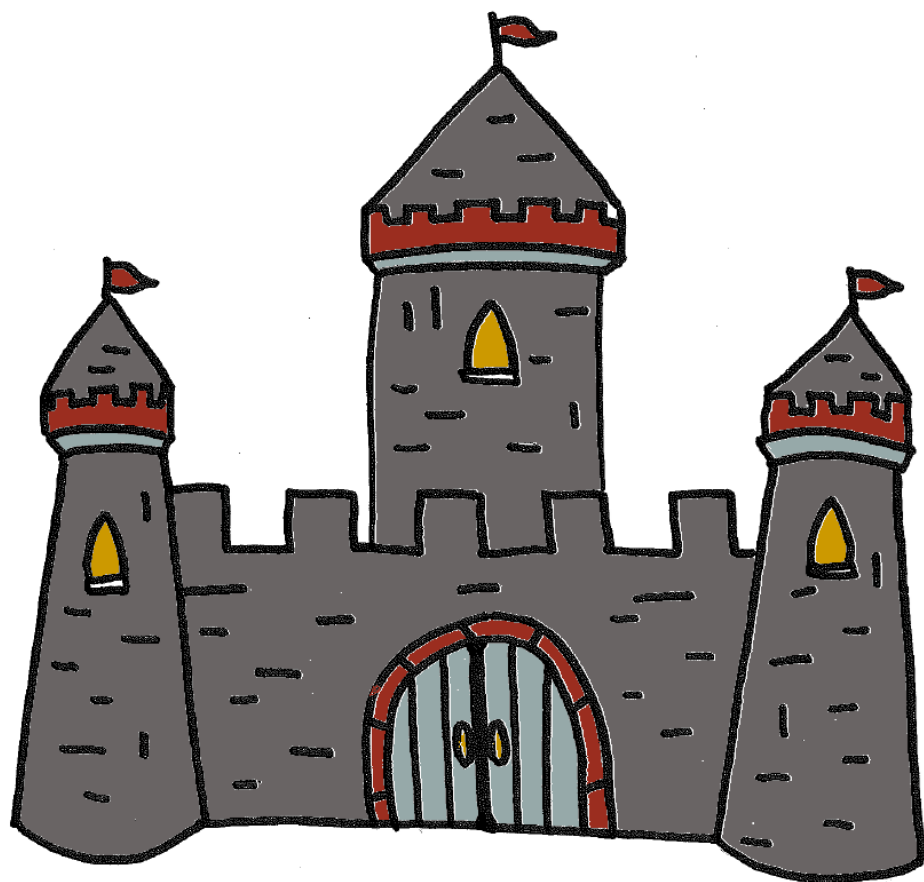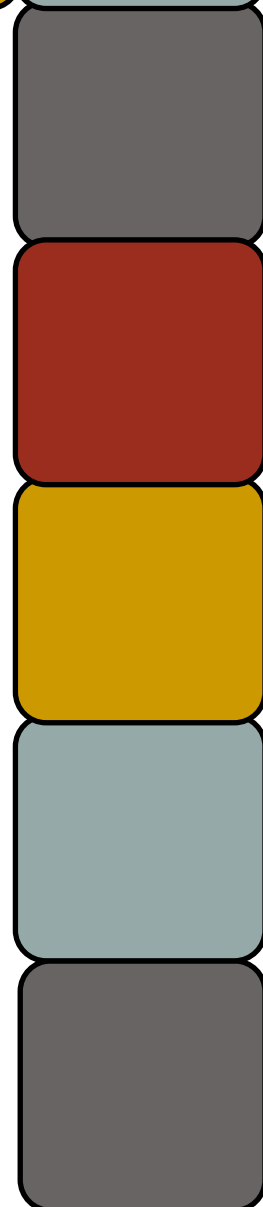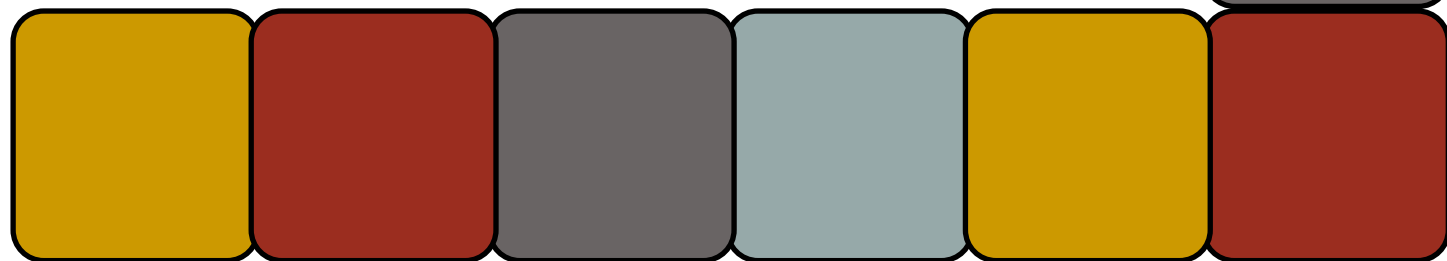

Supplement: Supplementary file 1 — RheumQuest Board.pdfRheumQuest Cards.pptxRheumQuest Instructions.docxFacilitator Guide.docxPre- and Posttest with Answer Key.docx [file mep_2374-8265.11587-s001.zip › A. RheumQuest Game Board.pdf]
